# Real-World Effectiveness of Influenza Vaccination Against Medically Attended Influenza and Severe Outcomes Among Older Adults in Yichang, China: A Multi-Season Population-Based Cohort Study

**DOI:** 10.3390/vaccines14100858

**Published:** 2026-09-28

**Authors:** Guanqi Wang, Hong Cao, Yi Yan, Lei Wang, Yangfan Sun, Da Feng

**Affiliations:** 1 School of Pharmacy, Tongji Medical College, Huazhong University of Science and Technology, Wuhan 430030, China; u7718024@anu.edu.au (G.W.); ch19970617@126.com (H.C.); 2 Institute of Immunization Planning, Yichang Center for Disease Control and Prevention, Yichang 443000, China; 3 Institute for Infectious Disease Control and Prevention, Hubei Provincial Center for Disease Control and Prevention, Wuhan 430079, China; 4 Wenlan School of Business, Zhongnan University of Economics and Law, Wuhan 430073, China

**Keywords:** influenza vaccine, vaccine effectiveness, older adults, real-world evidence, retrospective cohort, overlap weighting, hospitalization, China

## Abstract

**Background/Objectives:** Real-world evidence on influenza vaccine effectiveness (VE) against severe outcomes among older adults in China remains limited. We evaluated influenza vaccination against medically attended influenza, influenza hospitalization, and influenza-related intensive care unit (ICU) admission among adults aged ≥60 years in Yichang, China. **Methods:** We conducted a multi-season population-based retrospective cohort study using linked immunization and healthcare records. Season-specific propensity scores and overlap weighting were used to improve comparability between vaccinated and unvaccinated participants. Weighted Cox regression with individual-level clustered robust variance was used to estimate VE; proportional-hazards assumptions were formally assessed, with extended Cox models fitted when treatment-specific non-proportionality was detected. **Results:** The cohort comprised 1,106,886 person-seasons, including 31,540 vaccinated and 1,075,346 unvaccinated person-seasons. Vaccination was associated with lower hazards of medically attended influenza (VE, 55.7%; 95% CI, 43.4–65.3%), influenza hospitalization (54.4%; 39.4–65.7%), and influenza-related ICU admission (52.7%; 14.2–73.9%). Protective associations were observed in both winter seasons and persisted when the vaccination window was restricted to 14–180 days before the index date, with VE estimates of 78.4% for medically attended influenza and 83.1% for influenza hospitalization; findings were also robust across other sensitivity analyses. VE point estimates were numerically higher during higher antigenic-concordance periods, although the interaction was not statistically significant. **Conclusions:** Influenza vaccination within the prespecified pre-index exposure window was associated with lower risks of medically attended influenza and severe influenza-related outcomes among older adults in Yichang. Residual confounding and outcome misclassification cannot be excluded, and the estimates primarily apply to the overlap population rather than all older adults in Yichang.

## 1. Introduction

Seasonal influenza remains an important cause of morbidity and mortality worldwide, with an estimated 3–5 million cases of severe disease and approximately 290,000–650,000 influenza-associated respiratory deaths occurring annually [1]. The burden is disproportionately concentrated among older adults: global modeling suggests that influenza-associated respiratory mortality rises sharply with age, with the highest mortality rates observed among adults aged ≥75 years [2]. This vulnerability reflects not only age-related immune senescence but also the high prevalence of multimorbidity and diminished physiological reserve in older populations, which increase the likelihood that influenza progresses from an acute respiratory infection to pneumonia, exacerbation of underlying cardiopulmonary disease, hospitalization, functional decline, and death [3,4]. Influenza may also precipitate important extra-respiratory complications, including acute cardiovascular and cerebrovascular events, further amplifying its clinical burden in later life [3]. Consequently, the value of influenza vaccination in older adults extends beyond preventing medically attended infection alone; preventing progression to hospitalization and other severe clinical outcomes represents an equally important objective of seasonal vaccination programs [3,4].

Although seasonal influenza vaccination is recommended as a key preventive strategy for older adults, its effectiveness in routine practice is not constant and may vary substantially across populations and influenza seasons. Vaccine effectiveness (VE) reflects the combined influence of host characteristics, circulating viral strains, antigenic match between vaccine and circulating viruses, vaccine formulation, and the timing of vaccination relative to influenza exposure [5,6]. These sources of heterogeneity are particularly relevant in older populations, in whom immunosenescence and age-related changes in immune responsiveness may attenuate vaccine-induced protection [6]. A recent systematic review and meta-analysis of 191 test-negative design studies estimated an overall seasonal influenza VE of 41.4%, but found substantial variation by viral subtype, ranging from 55.4% against A(H1N1) to 26.8% against A(H3N2); pooled VE against any influenza was also lower among adults aged ≥65 years than among younger populations [5]. Moreover, evidence from studies of older adults indicates that VE estimates can vary across influenza seasons and according to the clinical outcome evaluated [5,7]. Consequently, vaccine efficacy demonstrated under controlled conditions may not translate directly into a uniform level of protection in routine clinical practice, underscoring the importance of contemporary real-world evaluations in specific populations and epidemiological settings [6,7].

In China, these uncertainties are compounded by persistently low influenza vaccination coverage and a comparatively limited evidence base on vaccine effectiveness among older adults. A recent systematic review and meta-analysis of 65 studies involving more than 149 million adults aged ≥60 years estimated a pooled influenza vaccination rate of only 17%, with even lower uptake in settings without free vaccination policies and among individuals with chronic diseases [8]. Influenza vaccine is not included in China’s National Immunization Program and is generally administered on a voluntary, self-paid basis, although some local governments have introduced publicly funded vaccination programs for selected high-risk populations, including older adults. National policy has consistently identified older adults as a priority group for annual influenza vaccination and has encouraged local governments with sufficient resources to provide free or subsidized vaccination [9]. More importantly, evidence on effectiveness remains considerably less extensive than that on vaccine uptake: only 14 studies comprising 13,950 participants contributed estimates against laboratory-confirmed influenza, yielding a pooled VE of 33%, with substantial heterogeneity across viral subtypes and vaccine–virus match [8]. An earlier meta-analysis similarly reported lower VE among adults aged ≥60 years than among younger populations and highlighted the need for continued assessment of influenza vaccine performance in China [10].

More recent studies have expanded the evidence base specifically among older adults. A four-season test-negative study in Ningbo among adults aged ≥60 years evaluated influenza vaccination from 2018–2019 through 2021–2022 and found no evidence that vaccination in consecutive seasons significantly reduced the protection associated with current-season vaccination, supporting continued annual vaccination in older adults [11]. A three-season study in northeastern Zhejiang during 2021–2024 estimated an overall VE of 47.2% against laboratory-confirmed influenza among older adults presenting with influenza-like illness while demonstrating substantial age-related heterogeneity, with VE declining from 70.3% among adults aged 60–69 years to 38.3% among those aged ≥80 years [12]. More recently, another three-season test-negative study in Jiaxing during 2022–2025 reported an adjusted overall VE of 42.5% and further demonstrated variation according to influenza season, viral subtype, underlying disease, and time since vaccination [13].

Collectively, these studies provide evidence that seasonal influenza vaccination confers meaningful but heterogeneous protection among older adults in China. However, the available evidence remains dominated by test-negative studies focused on laboratory-confirmed influenza and outpatient disease. Evidence regarding more clinically consequential outcomes, particularly influenza-related hospitalization and critical-care use, remains comparatively sparse, and these outcomes have rarely been evaluated together within the same longitudinal older-adult population. Consequently, large-scale multi-season studies that simultaneously assess influenza-related healthcare encounters, hospitalization, and critical-care outcomes—and rigorously address baseline differences between vaccinated and unvaccinated older adults—are needed to provide a more comprehensive characterization of the real-world protection afforded by seasonal influenza vaccination in China.

**Figure 1:**
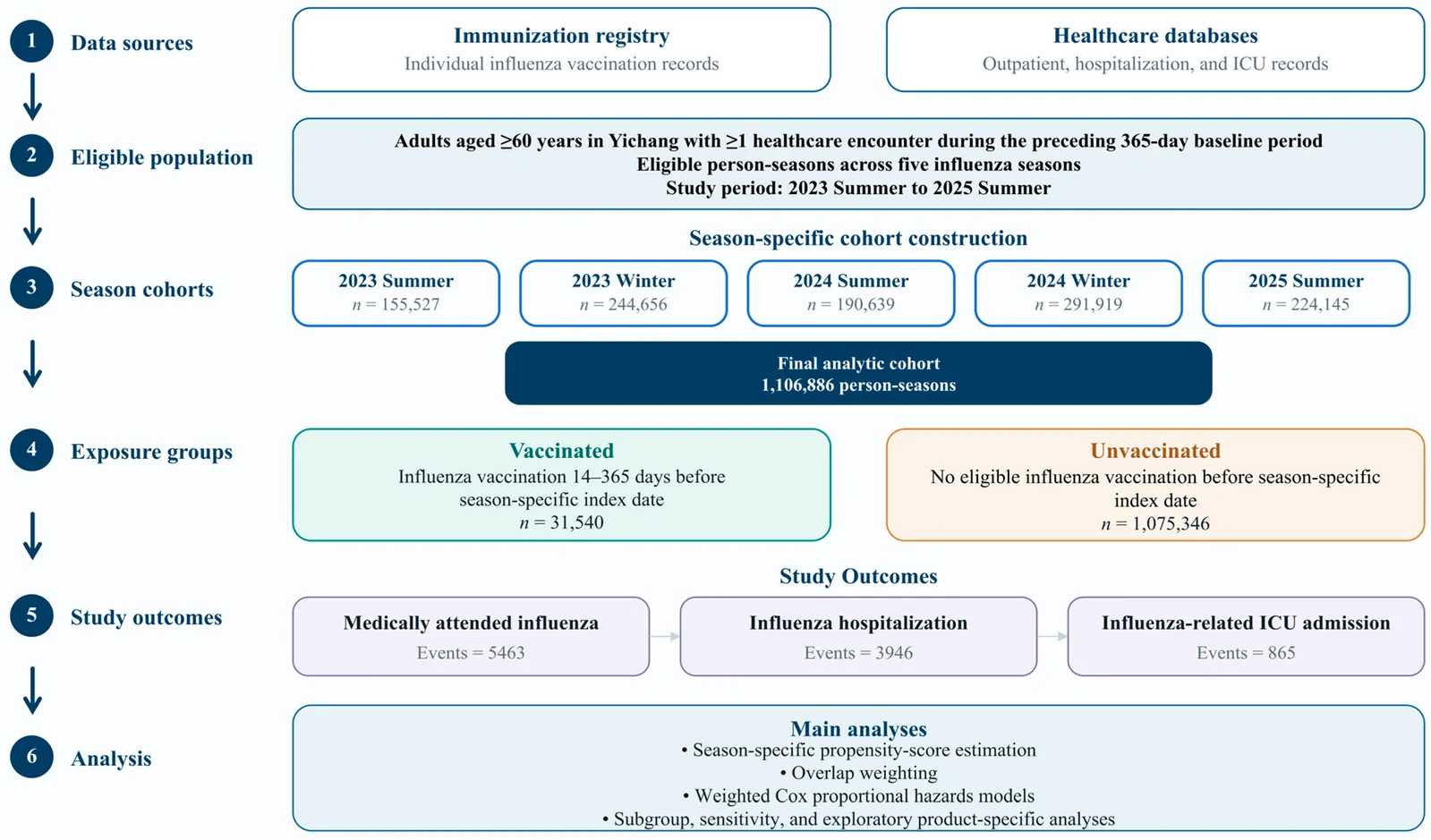
Study design and analytical workflow. **Note:** Blue-toned rectangles indicate data sources, cohort construction, and analytical procedures; green and orange rectangles indicate the vaccinated and unvaccinated exposure groups, respectively; purple rectangles indicate study outcomes. Numbered circles and vertical arrows indicate the sequential stages of the study workflow. Horizontal arrows between the study outcomes indicate increasing clinical severity and do not imply individual-level progression between outcomes. *n* denotes the number of person-seasons. ICU, intensive care unit.

To address these evidence gaps, we conducted a large, multi-season retrospective cohort study using linked individual-level vaccination and healthcare records from adults aged ≥60 years in Yichang, China. We constructed season-specific person-season cohorts with eligibility, vaccination status, and follow-up aligned to a common index date, and used season-specific propensity scores with overlap weighting to improve comparability between vaccinated and unvaccinated older adults. The primary objective was to estimate the real-world effectiveness of recorded influenza vaccination against three outcomes of increasing clinical severity: medically attended influenza, influenza hospitalization, and influenza-related intensive care unit admission. We further examined whether vaccine effectiveness varied across influenza seasons and according to age, sex, and chronic disease burden, with additional exploratory analyses by vaccine manufacturer and valency. To assess the robustness of the primary findings, we additionally conducted sensitivity analyses addressing vaccination timing, restriction to winter seasons, repeat vaccination history, and season-level vaccine–virus antigenic concordance. An overview of the study design and analytical workflow is presented in Figure 1. By evaluating these outcomes within a common longitudinal framework, this study aimed to provide a more comprehensive assessment of the real-world protection associated with influenza vaccination among older adults in China.

## 2. Materials and Methods

### 2.1. Study Design and Data Sources

We conducted a retrospective, multi-season cohort study among adults aged ≥60 years in Yichang, Hubei Province, China, following the Strengthening the Reporting of Observational Studies in Epidemiology (STROBE) recommendations [14]. Individual-level data were obtained from routinely collected healthcare and immunization records and included outpatient diagnostic records, inpatient diagnostic records, outpatient electronic medical records, and vaccination records. Records across data sources were linked at the individual level using anonymized identifiers together with demographic information.

The analysis was structured as a person-season cohort, allowing eligible individuals to contribute observations to more than one influenza season. Summer seasons were defined from 1 May to 31 July and winter seasons from 1 October to 31 March of the following year. These prespecified analytical windows were selected to capture recurrent periods of elevated summer and winter–spring influenza activity in Yichang and Hubei rather than to imply an absence of influenza circulation outside these intervals. Previous laboratory-based surveillance in Yichang has demonstrated complex annual, semiannual, and year-round influenza circulation patterns, including both summer and winter–spring epidemics, while longer-term surveillance in Hubei Province has similarly documented pronounced winter activity with occasional summer increases [15,16,17,18]. The season-specific index date was defined as the first day of each influenza season. Only seasons with complete outcome follow-up within the available data period were included. For each season, eligibility was determined at the season-specific index date. Individuals were required to be aged ≥60 years at the season-specific index date and to have at least one healthcare encounter during the 365-day baseline period preceding the index date. Individuals with prespecified immunocompromising conditions identified during the baseline period were excluded. The resulting eligible population was followed from the season-specific index date until the occurrence of the outcome of interest or the applicable censoring date, as described below.

### 2.2. Influenza Vaccination Exposure

Influenza vaccination status was determined from individual-level vaccination records for each season-specific index date. Participants were classified as vaccinated if they had received an influenza vaccine 14–365 days before the index date, with the most recent eligible vaccination used when more than one vaccination record was available. The 14-day lower bound was applied because protective immune responses generally require approximately two weeks to develop after influenza vaccination, whereas the 365-day upper bound was used as a pragmatic primary exposure window to capture influenza vaccination during the preceding year, consistent with the recommendation for annual seasonal influenza vaccination [19]. Individuals vaccinated within 0–13 days before the index date were excluded from the primary exposure comparison.

Participants without an eligible influenza vaccination before the index date were classified as unvaccinated. Vaccinated and unvaccinated participants entered follow-up on the same season-specific index date. If an initially unvaccinated participant received an influenza vaccine during follow-up, observation was censored on the vaccination date. The primary exposure window was 14–365 days before the index date; more restrictive 14–90-day, 14–180-day, and 14–270-day exposure windows were examined in sensitivity analyses. Although August and September were not included in the prespecified influenza follow-up windows, records occurring during these months were not discarded. Influenza vaccinations administered during August–September could contribute to vaccination classification for a subsequent season if they fell within the corresponding 14–365-day pre-index exposure window. Likewise, healthcare encounters and other baseline covariate information recorded during August–September could contribute to the 365-day baseline period preceding a subsequent season-specific index date. Individual vaccination dates were available for all eligible vaccination records and were used to characterize vaccination timing relative to each season-specific index date. However, lot-specific antigenic composition was not available for all vaccination records; therefore, correspondence between each administered vaccine and a specific seasonal vaccine formulation could not be directly verified. Because censoring at subsequent vaccination could be informative if vaccination timing were related to evolving health status or healthcare utilization, we additionally evaluated vaccination as a time-varying exposure in a sensitivity analysis, as described in Section 2.5.

### 2.3. Outcomes and Baseline Covariates

Three prespecified outcomes were evaluated during each influenza season: medically attended influenza diagnosis, hospitalization with an influenza diagnosis, and influenza-related intensive care unit (ICU) admission. Medically attended influenza diagnosis was defined as the first qualifying influenza diagnosis recorded during either an outpatient or inpatient healthcare encounter. Hospitalization with an influenza diagnosis was defined as the first inpatient encounter during which a qualifying influenza diagnosis was recorded. The analytic dataset did not contain reliable diagnosis-position fields; therefore, primary and secondary influenza diagnoses could not be distinguished. Likewise, emergency-department encounters could not be reliably distinguished from other outpatient encounters in the available analytic data. The ICD-10-based outcome algorithm was not independently validated against chart review or systematic laboratory-confirmation data within the Yichang dataset. Complete code definitions are provided in Table A1, and outcome specificity was additionally evaluated using the J10-restricted sensitivity analysis described in Section 2.5. Hospitalization is a well-established indicator of severe influenza-associated illness in influenza surveillance and vaccine-effectiveness research [20,21]. For each outcome, only the first qualifying event during a given person-season was considered.

Influenza-related ICU admission was defined by temporal linkage between an ICU record and a qualifying influenza diagnosis. An ICU admission was considered influenza-associated when it occurred from 1 day before through 14 days after the first qualifying influenza diagnosis. The 14-day attribution window was selected a priori to capture critical-care episodes occurring in close temporal proximity to the influenza diagnosis and was informed by established influenza surveillance frameworks that use a 14-day window to temporally link influenza infection with hospitalization [21]. ICU admission is also commonly evaluated as a severe influenza-associated outcome in vaccine-effectiveness studies [22]. This operational definition was intended to identify severe illness temporally associated with an influenza diagnosis rather than to establish direct etiologic attribution to influenza.

Baseline covariates were assessed during the 365 days preceding each season-specific index date. Demographic variables included age and sex. Prespecified chronic conditions included hypertension, diabetes mellitus, chronic respiratory disease, ischemic heart disease, heart failure, cerebrovascular disease, chronic kidney disease, cancer, dementia, chronic liver disease, and tuberculosis. Chronic conditions were identified from pre-index diagnostic records using prespecified ICD-10 algorithms, and the total number of chronic conditions was used to characterize chronic disease burden. The complete ICD-10 code definitions used for influenza outcomes, baseline comorbidities, and prespecified immunocompromising exclusion conditions are provided in Table A1.

Baseline healthcare utilization was measured as the number of unique days with outpatient visits, inpatient admissions, and any healthcare contact during the baseline period. Previous influenza diagnoses, prior pneumococcal vaccination, and receipt of other non-influenza vaccines during the baseline period were also assessed when data were available. The “other vaccination” variable captured receipt of any non-influenza vaccine recorded in the source database. These variables were included to characterize differences in baseline health status, healthcare utilization, and preventive-health behavior between vaccinated and unvaccinated participants.

### 2.4. Propensity Score Weighting and Covariate Balance

Propensity scores (PSs) were estimated separately within each influenza season using multivariable logistic regression, with influenza vaccination status as the dependent variable. The model included age, sex, hypertension, diabetes mellitus, chronic respiratory disease, ischemic heart disease, heart failure, cerebrovascular disease, chronic kidney disease, cancer, dementia, chronic liver disease, tuberculosis, baseline outpatient, inpatient, and total healthcare utilization, prior influenza diagnosis, prior pneumococcal vaccination, and receipt of other vaccines during the baseline period. Age was modeled using a natural cubic spline with three degrees of freedom, and healthcare-utilization measures were log-transformed to reduce the influence of highly skewed values.

Overlap weighting was used as the primary propensity-score method [23,24,25]. Vaccinated participants were assigned a weight of (1-PS), whereas unvaccinated participants were assigned a weight of (PS). This weighting scheme emphasizes participants with the greatest overlap in their predicted probabilities of vaccination and targets the average treatment effect in the overlap population.

To assess propensity-score overlap and the empirical positivity assumption, propensity-score distributions were examined separately for vaccinated and unvaccinated person-seasons within each influenza season. Common support was defined as the intersection of the observed propensity-score ranges in the two exposure groups. We additionally calculated the proportions of vaccinated and unvaccinated person-seasons falling outside the common-support region and the effective sample size (ESS) after overlap weighting, using $ESS=\;\frac{{\left(\sum w_{i}\right)}^{2}}{\sum w_{i}}$. These diagnostics were evaluated separately within each influenza season. The common-support assessment was used as a diagnostic rather than as an exclusion criterion. No observations were excluded solely because their propensity scores fell outside the empirical common-support interval; all otherwise eligible observations were retained in the primary overlap-weighted analyses. Overlap weighting continuously down-weights observations with propensity scores near the extremes rather than requiring an arbitrary trimming threshold.

Covariate balance before and after weighting was evaluated using absolute standardized mean differences (SMDs) [26]. An absolute SMD < 0.10 was considered indicative of adequate balance. Covariate balance was additionally assessed within each influenza season to evaluate the performance of the weighting procedure.

### 2.5. Statistical Analysis

Time-to-event associations between influenza vaccination and each outcome of interest were estimated using weighted Cox proportional hazards regression [27]. Observation began on the season-specific index date and continued until the first occurrence of the outcome of interest or the applicable censoring date. Concurrent mortality data and individual-level dates of death were not available in the linked healthcare and immunization datasets. Therefore, non-influenza death could not be explicitly treated as a competing event or incorporated into a competing-risks analysis. Primary models incorporated overlap weights and additionally adjusted for the baseline covariates included in the propensity-score model. The additional outcome-model covariate adjustment was intended to improve precision by accounting for prognostic baseline variation after weighting. To assess whether the estimated associations depended on this additional adjustment, we conducted a specification sensitivity analysis in which the models were refitted using overlap weights alone, without additional baseline covariate adjustment. Influenza season was treated as a stratification factor, allowing the baseline hazard to vary across seasons. Because the same individuals could be included in more than one influenza season, robust variance estimates were clustered at the individual level [28]. Tied event times were handled using the Efron method. The proportional hazards assumption for the vaccination effect was assessed using tests based on scaled Schoenfeld residuals [29]. When evidence of treatment-specific non-proportionality was identified, an extended Cox model was fitted to allow the association between vaccination and the outcome to vary over follow-up time [30]. For these outcomes, follow-up was partitioned at 90 days, and separate vaccination hazard ratios were estimated for the periods ≤90 days and >90 days. Hazard ratios (HRs) with 95% confidence intervals (CIs) were estimated, and vaccine effectiveness (VE) was calculated as $VE=\left(1-HR\right)\times 100\%$ [31].

Season-specific analyses were conducted by fitting the weighted Cox model separately within each influenza season. Prespecified subgroup analyses for medically attended influenza were performed according to age group (60–69, 70–79, and ≥80 years), sex, and chronic disease burden (0–1 versus ≥2 conditions). Effect modification was assessed using vaccination-by-subgroup interaction terms, with joint interaction tests used for subgroup variables with more than two levels.

Records with insufficient information for ascertainment of the prespecified study outcomes were excluded from the analytic dataset. No imputation was performed for baseline covariates. The “Unknown” category for sex was an explicit category in the source database and was retained as recorded rather than treated as missing or imputed. Its relatively high frequency reflects incomplete demographic information in the underlying routine administrative records rather than loss of information during study processing. Sex was entered into the propensity-score model as a categorical variable, with “Unknown” retained as a separate category. For the exploratory product-specific analyses, manufacturer and valency information was unavailable for some vaccination records because these fields were not provided in the source database; these analyses were therefore restricted to person-seasons with available product information. No adjustment for multiple comparisons was applied to the exploratory subgroup or product-specific analyses; these results were therefore interpreted as hypothesis-generating.

Several sensitivity analyses were conducted to assess the robustness of the primary estimates. First, to assess the potential for informative censoring among participants who were unvaccinated at the season-specific index date but subsequently received influenza vaccination during follow-up, we quantified the number and proportion of such person-seasons within each influenza season and compared their baseline characteristics with those of participants who remained unvaccinated throughout follow-up using standardized mean differences. We then performed a time-varying exposure sensitivity analysis. Participants vaccinated before the index date were considered exposed from the start of follow-up, whereas initially unvaccinated participants contributed unvaccinated person-time until 14 days after subsequent vaccination and vaccinated person-time thereafter. Time-varying Cox models were adjusted for the prespecified baseline covariates, stratified by influenza season, and used robust variance estimation clustered at the individual level. The time-varying exposure model adjusted only for the same prespecified baseline covariates used in the primary analysis. Health status, healthcare utilization, and other factors potentially associated with subsequent vaccination were not incorporated as time-updated covariates during follow-up; therefore, this sensitivity analysis did not account for time-dependent confounding arising from factors that evolved after the index date. Second, the vaccination exposure window was progressively restricted from the primary 14–365-day definition to 14–90, 14–180, and 14–270 days before the index date. For each alternative exposure definition, propensity scores and overlap weights were re-estimated separately within each influenza season. Third, analyses were restricted to winter influenza seasons. Fourth, vaccinated person-seasons with more than one influenza vaccination record within the preceding 365 days were excluded, after which season-specific propensity scores and overlap weights were re-estimated. Fifth, a season-level antigenic-concordance sensitivity analysis was conducted using influenza surveillance data on predominant circulating subtypes and the proportion of antigenically characterized viruses similar to the corresponding vaccine strain. The 2024 summer and 2024 winter periods were classified as higher-concordance seasons, whereas the 2023 winter period was classified as a lower-concordance season. Propensity scores and overlap weights were re-estimated within each included season. Because antigenic concordance was defined at the season level and only three study periods contributed to this comparison, the concordance analysis and vaccination-by-concordance interaction test were considered exploratory. Sixth, to assess the robustness of the findings to outcome specificity, we conducted a sensitivity analysis restricting influenza outcomes to ICD-10 J10-coded events, which denote influenza due to an identified influenza virus. The same overlap-weighted Cox modeling framework with additional adjustment for the prespecified baseline covariates, stratification by influenza season, and individual-level clustered robust variance estimation was applied to J10-coded medically attended influenza and J10-coded influenza hospitalization.

Exploratory manufacturer- and valency-specific analyses were conducted for medically attended influenza by comparing each eligible vaccine product separately with contemporaneous unvaccinated person-seasons. For each product-specific comparison, propensity scores and overlap weights were re-estimated separately within each influenza season. These analyses were exploratory and were not interpreted as head-to-head comparisons between vaccine products. All statistical tests were two-sided, with *p* < 0.05 considered statistically significant. Statistical analyses were performed using R version 4.6.1 (R Foundation for Statistical Computing, Vienna, Austria), primarily with the data.table (version 1.18.4), survival (version 3.8-6), cobalt (version 4.6.3), ggplot2 (version 4.0.3), and splines (version 4.6.1) packages.

## 3. Results

### 3.1. Study Population and Covariate Balance

The final analytic cohort comprised 1,106,886 person-seasons contributed by 641,747 unique individuals, including 31,540 vaccinated and 1,075,346 unvaccinated person-seasons (Table 1). Median follow-up was 91.5 days (IQR, 91.5–181.5 days) across the three primary outcome analyses. Before weighting, vaccinated participants were older and had a higher prevalence of several chronic conditions than the unvaccinated participants. They also had greater baseline healthcare utilization and were more likely to have received other vaccines. The largest baseline imbalances were observed for prior receipt of other vaccines, pneumococcal vaccination, healthcare utilization, and chronic disease burden. Among the 26,490 unique influenza vaccination records contributing to the analytic dataset, 5624 (21.23%) occurred during August or September. These records were retained and could contribute to vaccination classification for a subsequent influenza season when they fell within the prespecified 14–365-day pre-index exposure window.

**Figure A1:**
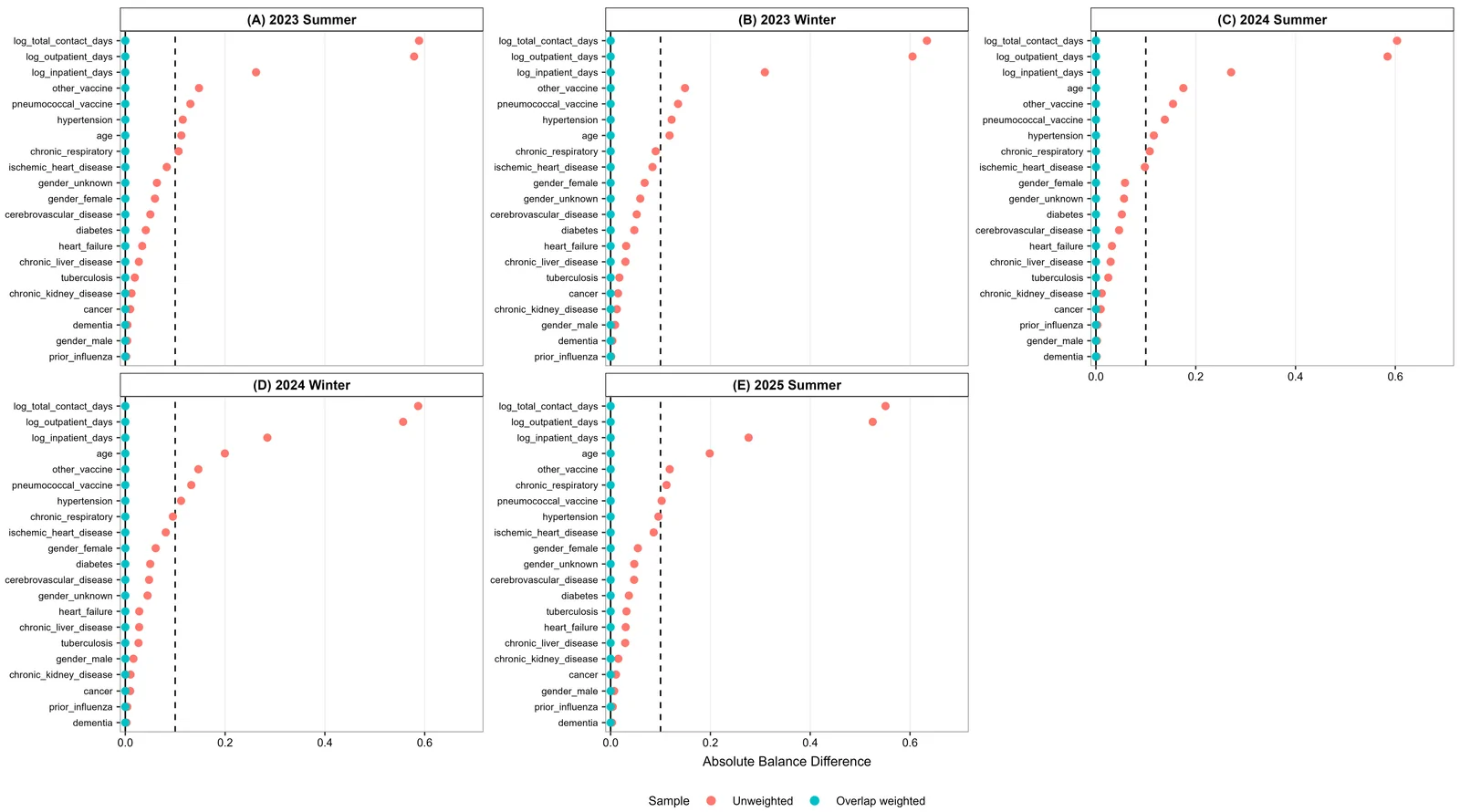
Season-specific covariate balance before and after overlap weighting. **Note:** Red points represent unweighted balance differences, and blue-green points represent balance differences after overlap weighting. For continuous covariates, balance is expressed as the absolute standardized mean difference; for binary covariates, balance is expressed as the absolute difference in proportions. The solid vertical line at 0 indicates no between-group difference, and the dashed vertical line at 0.10 indicates the prespecified threshold for adequate covariate balance.

Overlap weighting substantially improved comparability between the vaccination groups. After weighting, all reported absolute standardized mean differences were ≤0.050. Season-specific balance diagnostics showed similarly good covariate balance after weighting across each influenza season, as illustrated in Figure A1.

**Figure 2:**
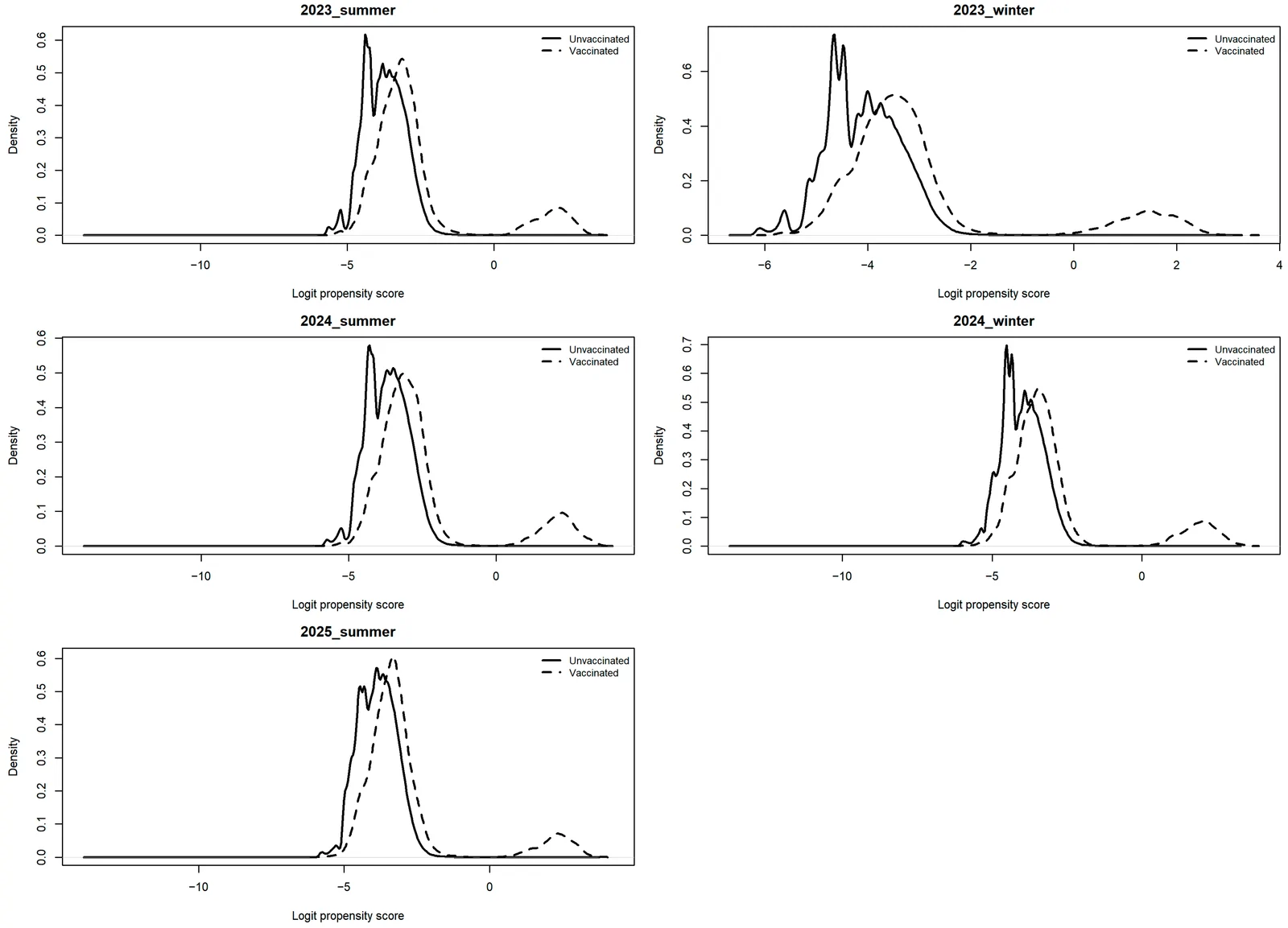
Season-specific distributions of logit-transformed propensity scores according to influenza vaccination status. **Note:** Solid and dashed curves represent unvaccinated and vaccinated person-seasons, respectively. Propensity scores were estimated separately within each influenza season using the prespecified baseline covariates. The distributions showed substantial empirical overlap across all five influenza seasons, consistent with the common-support diagnostics reported in Table 2.

Propensity-score diagnostics further indicated adequate empirical overlap between vaccinated and unvaccinated person-seasons across all five influenza seasons. Common support was present in every season. The proportions of observations outside the empirical common-support region ranged from 0.07% to 0.82% among unvaccinated person-seasons and from 0.00% to 0.20% among vaccinated person-seasons. The unvaccinated-group ESS ranged from 72,210 to 125,502 across seasons, whereas the vaccinated-group ESS ranged from 4622 to 6702. The corresponding overall overlap-weighted ESS ranged from 17,448 to 25,477. Although small proportions of observations fell outside the empirical common-support interval, these observations were retained in the overlap-weighted analyses rather than excluded by trimming. Detailed season-specific diagnostics are presented in Table 2, and the corresponding propensity-score distributions are shown in Figure 2.

### 3.2. Primary Vaccine Effectiveness

Table 3 summarizes the primary estimates of influenza vaccine effectiveness across the three outcomes. During follow-up, 5463 medically attended influenza diagnoses, 3946 hospitalizations with an influenza diagnosis, and 865 influenza-related ICU admissions were identified. In the overlap-weighted Cox models, influenza vaccination was associated with a lower hazard of all three outcomes. For medically attended influenza diagnosis, the HR was 0.443 (95% CI, 0.347–0.566), corresponding to a VE of 55.7% (95% CI, 43.4–65.3%; *p* < 0.001). For hospitalization with an influenza diagnosis, the HR was 0.456 (95% CI, 0.343–0.606), corresponding to a VE of 54.4% (95% CI, 39.4–65.7%; *p* < 0.001). For influenza-related ICU admission, the HR was 0.473 (95% CI, 0.261–0.858), corresponding to a VE of 52.7% (95% CI, 14.2–73.9%; *p* = 0.014). The 3946 influenza-hospitalization events represented approximately 72% of the 5463 medically attended influenza events. Because medically attended influenza and influenza hospitalization were prespecified, overlapping clinical endpoints rather than mutually exclusive categories, this proportion should not be interpreted as a population-level hospitalization rate among all influenza infections.

In the model-specification sensitivity analysis, estimates from models using overlap weights alone were highly similar to those from the primary overlap-weighted models with additional baseline covariate adjustment. The corresponding HRs from the overlap-weighted-only models were 0.445 for medically attended influenza, 0.468 for influenza hospitalization, and 0.483 for influenza-related ICU admission compared with 0.443, 0.456, and 0.473, respectively, in the primary models. These findings indicate that the estimated associations were not materially dependent on the additional outcome-model covariate adjustment.

Assessment of the proportional hazards assumption using scaled Schoenfeld residuals indicated treatment-specific non-proportionality for medically attended influenza and influenza hospitalization (both *p* < 0.001), whereas the proportional hazards assumption was not rejected for influenza-related ICU admission (*p* = 0.159). Accordingly, extended Cox models were fitted for medically attended influenza and influenza hospitalization. For medically attended influenza, the vaccination HR was 0.101 (95% CI, 0.045–0.225) during the first 90 days of follow-up and 0.469 (95% CI, 0.355–0.620) thereafter. For influenza hospitalization, the corresponding HRs were 0.076 (95% CI, 0.025–0.237) and 0.490 (95% CI, 0.358–0.670), respectively. Thus, for both outcomes, the magnitude of the protective association varied over follow-up, with stronger associations during the first 90 days and persistent protective associations thereafter. The single Cox HRs for these two outcomes are therefore presented as summary associations over the observed follow-up rather than as constant effects applied uniformly throughout the risk period (Table 3).

To complement the relative effect estimates from the overlap-weighted Cox models, we additionally examined the absolute event risks after overlap weighting. As shown in Table 4, the weighted event risks were consistently lower among vaccinated than unvaccinated person-seasons across all three outcomes. For medically attended influenza, the weighted event risk was 1.92 versus 5.58 per 1000 person-seasons in the vaccinated and unvaccinated participants, respectively. Corresponding weighted risks were 1.47 versus 4.07 per 1000 person-seasons for influenza hospitalization and 0.33 versus 1.09 per 1000 person-seasons for influenza-related ICU admission. These absolute-risk estimates were directionally consistent with the protective associations observed in the weighted Cox analyses.

### 3.3. Season-Specific Vaccine Effectiveness

Influenza vaccine effectiveness can vary across seasons according to circulating viral strains, vaccine–virus match, population characteristics, and local epidemiological conditions [5,8,12,32,33]. Recent studies from Beijing, Zhejiang, and Wuhan have similarly reported variability in VE across influenza seasons, viral subtypes, and older-adult populations in China [12,32,33]. Table 5 summarizes the season-specific estimates in the present study. During the 2023 winter season, influenza vaccination was associated with lower hazards of medically attended influenza (HR, 0.551; 95% CI, 0.382–0.795; VE, 44.9%; *p* = 0.001) and influenza hospitalization (HR, 0.601; 95% CI, 0.398–0.907; VE, 39.9%; *p* = 0.015). The estimate for influenza-related ICU admission was also protective in direction but less precise (HR, 0.588; 95% CI, 0.219–1.577; VE, 41.2%; *p* = 0.291).

Similar associations were observed during the 2024 winter season, with VE estimates of 50.9% for medically attended influenza (HR, 0.491; 95% CI, 0.350–0.689; *p* < 0.001) and 56.6% for influenza hospitalization (HR, 0.434; 95% CI, 0.288–0.655; *p* < 0.001). For influenza-related ICU admission, the VE estimate was 53.2%, although the confidence interval included the null (HR, 0.468; 95% CI, 0.209–1.047; *p* = 0.065). During the 2024 summer season, larger point estimates were observed for medically attended influenza (VE, 87.3%) and influenza hospitalization (VE, 78.5%), although these estimates were less precise. These seasonal differences were also considered in the context of contemporaneous virological surveillance. The 2023 winter season was dominated by A(H3N2) viruses and showed lower antigenic concordance with the corresponding vaccine-like strain, whereas the 2024 summer and winter periods were dominated by A(H1N1) pdm09 viruses and showed substantially higher antigenic concordance. The virological characteristics underlying these season-level classifications are summarized in Table A3, and their association with VE was examined in the sensitivity analyses described in Section 3.5. Such seasonal variation is consistent with previous evidence showing that influenza VE may differ according to viral subtype and vaccine–circulating strain match [5,8,12].

Estimates for the 2023 and 2025 summer seasons were not estimable because very few or no outcome events occurred among the vaccinated participants, resulting in sparse-data separation in the Cox models. These estimates were therefore not interpreted as evidence of complete vaccine protection.

**Figure 3:**
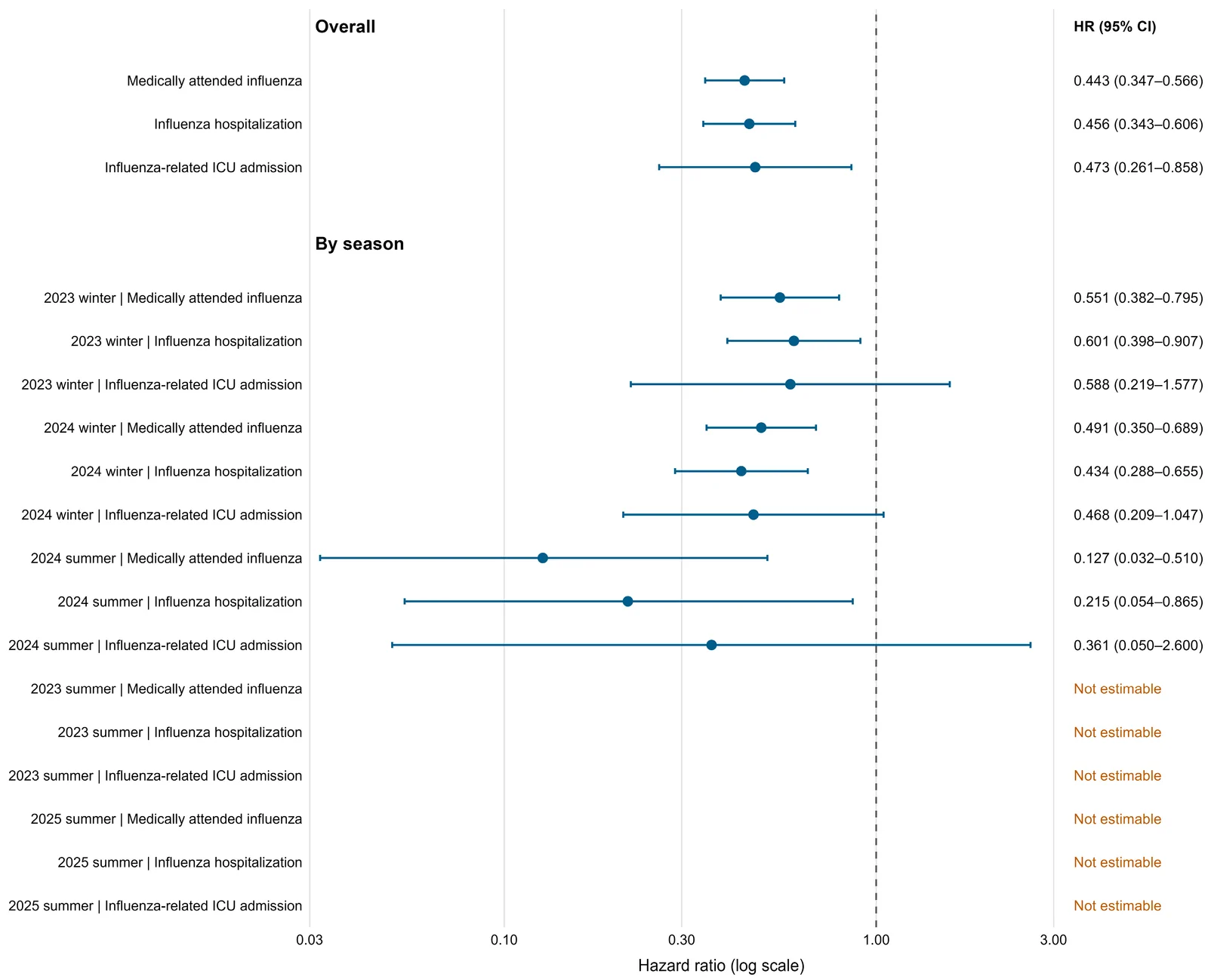
Overall and season–specific estimates of influenza vaccine effectiveness. **Note:** Points represent hazard ratio (HR) estimates, and horizontal blue lines represent the corresponding 95% confidence intervals. The vertical dashed line at HR = 1.00 indicates the null value. Estimates that could not be reliably obtained because of sparse outcome events are shown as “Not estimable”. HR, hazard ratio; CI, confidence interval.

Figure 3 visually summarizes the overall and season-specific hazard ratio estimates, demonstrating broadly consistent protective associations across the primary outcomes and the two winter influenza seasons. Overall, the season-specific analyses showed consistent protective associations for medically attended influenza and influenza hospitalization across both winter seasons, whereas estimates for ICU admission were less precise. Estimates for the summer seasons were also less precise and showed greater variability.

### 3.4. Subgroup Analyses

Age, sex, and underlying chronic conditions have been identified as potential modifiers of immune responses and influenza vaccine effectiveness in older adults [34,35,36,37]. Table 6 summarizes the prespecified subgroup analyses for medically attended influenza.

Subgroup analyses were restricted to medically attended influenza diagnosis because this outcome had the largest number of events and therefore provided the greatest statistical precision for evaluating potential effect modification. Corresponding subgroup analyses for hospitalization and ICU admission were not prespecified because the smaller numbers of severe outcome events, particularly ICU admissions, were expected to yield unstable stratum-specific estimates and limited power for interaction testing. Protective associations were observed across all three age groups, with VE estimates of 60.4% among adults aged 60–69 years (HR, 0.396; 95% CI, 0.263–0.596), 68.3% among those aged 70–79 years (HR, 0.317; 95% CI, 0.213–0.472), and 73.0% among those aged ≥80 years (HR, 0.270; 95% CI, 0.134–0.543). However, there was no evidence of effect modification by age group (*p* for interaction = 0.564).

The protective association was statistically significant in both sex strata. Among women, the estimated VE was 80.0% (HR, 0.200; 95% CI, 0.122–0.329) compared with 55.5% among men (HR, 0.445; 95% CI, 0.321–0.618). The vaccination-by-sex interaction reached nominal statistical significance (*p* for interaction = 0.045). Given the borderline interaction *p*-value and the multiple subgroup comparisons performed, this finding was considered exploratory.

Protective associations were similarly observed across levels of chronic disease burden. VE was 71.8% among participants with 0–1 chronic conditions (HR, 0.282; 95% CI, 0.187–0.424) and 61.0% among those with ≥2 chronic conditions (HR, 0.390; 95% CI, 0.276–0.552). There was no evidence of effect modification by chronic disease burden (*p* for interaction = 0.249). Overall, the subgroup analyses showed protective associations across age and chronic-disease strata, with no statistically supported heterogeneity except for the nominal sex interaction.

### 3.5. Sensitivity Analyses

Among the 1,075,346 person-seasons classified as unvaccinated at the season-specific index date, 7485 (0.70%) were subsequently vaccinated and censored during follow-up. The corresponding numbers were 41 (0.03%) in the 2023 summer season, 4370 (1.83%) in the 2023 winter season, 57 (0.03%) in the 2024 summer season, 2972 (1.04%) in the 2024 winter season, and 45 (0.02%) in the 2025 summer season. Most vaccination-related censoring therefore occurred during the two winter seasons. Baseline comparisons indicated meaningful differences in several characteristics, particularly healthcare utilization, chronic respiratory disease, chronic disease burden, and prior receipt of non-influenza vaccines, with several absolute standardized mean differences exceeding 0.10, as detailed in Appendix A.5 and Table A4.

In the time-varying exposure sensitivity analysis, in which initially unvaccinated participants contributed unvaccinated person-time until 14 days after subsequent vaccination and vaccinated person-time thereafter, the protective associations remained evident for all three outcomes. The HR was 0.371 (95% CI, 0.301–0.459) for medically attended influenza, 0.366 (95% CI, 0.286–0.469) for influenza hospitalization, and 0.276 (95% CI, 0.155–0.492) for influenza-related ICU admission, corresponding to VE estimates of 62.9%, 63.4%, and 72.4%, respectively. These findings indicate that the primary associations were not dependent on censoring participants at the time of subsequent vaccination.

Additional analyses were conducted to examine whether the primary estimates were robust to alternative vaccination exposure windows, restriction to winter influenza seasons, repeat influenza vaccination within the preceding 365 days, variation in season-level vaccine–virus antigenic concordance, and a more specific ICD-10 J10-coded outcome definition. Such analyses are particularly relevant in observational influenza vaccine-effectiveness studies because estimates may be influenced by vaccination timing, seasonal circulation patterns, vaccination history, vaccine–virus match, exposure classification, and residual confounding [11,31,38,39]. WHO guidance likewise emphasizes careful ascertainment of vaccination history and timing when interpreting observational influenza VE estimates [31].

The timing of influenza vaccination relative to the season-specific index date was also examined to characterize the primary 14–365-day exposure definition. Among the vaccinated person-seasons, 10.6% received vaccination 14–90 days before the index date, 21.0% at 91–180 days, 38.0% at 181–270 days, and 30.3% at 271–365 days. The distribution varied across influenza seasons, with more remote vaccination particularly common in the two winter seasons. Detailed season-specific distributions are presented in Appendix A.6 and Table A5. Among the additional sensitivity analyses, protective associations remained evident across the three more restrictive vaccination exposure windows for medically attended influenza and influenza hospitalization (Table 7). For medically attended influenza, VE estimates were highly consistent across the 14–90-day, 14–180-day, and 14–270-day windows, at 77.8% (HR, 0.222; 95% CI, 0.094–0.524; *p* < 0.001), 78.4% (HR, 0.216; 95% CI, 0.109–0.428; *p* < 0.001), and 79.6% (HR, 0.204; 95% CI, 0.123–0.338; *p* < 0.001), respectively. For influenza hospitalization, VE was 92.1% under the 14–90-day window (HR, 0.079; 95% CI, 0.015–0.409; *p* = 0.002), 83.1% under the 14–180-day window (HR, 0.169; 95% CI, 0.068–0.425; *p* < 0.001), and 78.4% under the 14–270-day window (HR, 0.216; 95% CI, 0.120–0.391; *p* < 0.001). Estimates for influenza-related ICU admission remained protective in direction across all three windows but were less precise under the 14–90-day and 14–180-day definitions; the corresponding VE estimates were 74.9%, 58.2%, and 65.1%, respectively, with the 14–270-day estimate reaching statistical significance (HR, 0.349; 95% CI, 0.142–0.860; *p* = 0.022).

Restricting the analysis to winter influenza seasons produced estimates similar in magnitude to the primary findings. VE was 60.7% for medically attended influenza (HR, 0.393; 95% CI, 0.301–0.514; *p* < 0.001), 60.4% for influenza hospitalization (HR, 0.396; 95% CI, 0.291–0.539; *p* < 0.001), and 65.6% for influenza-related ICU admission (HR, 0.344; 95% CI, 0.179–0.662; *p* = 0.001).

Repeat influenza vaccination within the preceding 365 days was uncommon. Among the 31,540 vaccinated person-seasons, 31,092 (98.58%) had one influenza vaccination record and 448 (1.42%) had two records, with no vaccinated person-season having three or more records. Repeat vaccination was concentrated primarily in the two winter seasons. After excluding these 448 person-seasons and re-estimating season-specific propensity scores and overlap weights, 1,106,438 person-seasons remained in the analysis. VE remained 66.1% for medically attended influenza (5461 events; HR, 0.339; 95% CI, 0.260–0.442; *p* < 0.001), 64.3% for influenza hospitalization (3945 events; HR, 0.357; 95% CI, 0.264–0.484; *p* < 0.001), and 70.2% for influenza-related ICU admission (865 events; HR, 0.298; 95% CI, 0.156–0.568; *p* < 0.001). These findings are consistent with previous evidence from Chinese older adults showing that repeated or consecutive-season influenza vaccination does not necessarily eliminate the protective effect of current-season vaccination [11].

In an additional sensitivity analysis incorporating season-level vaccine–virus antigenic concordance, the 2024 summer and 2024 winter periods were classified as higher-concordance seasons, whereas the 2023 winter period was classified as a lower-concordance season based on the predominant circulating subtype and the proportion of antigenically characterized viruses similar to the corresponding vaccine strain. After re-estimation of the season-specific propensity scores and overlap weights, VE during higher-concordance seasons was 69.7% for medically attended influenza (HR, 0.303; 95% CI, 0.212–0.434; *p* < 0.001), 71.7% for influenza hospitalization (HR, 0.283; 95% CI, 0.183–0.436; *p* < 0.001), and 74.2% for influenza-related ICU admission (HR, 0.258; 95% CI, 0.110–0.609; *p* = 0.002). Corresponding estimates during the lower-concordance season were 57.4% for medically attended influenza (HR, 0.426; 95% CI, 0.289–0.629; *p* < 0.001), 50.0% for influenza hospitalization (HR, 0.500; 95% CI, 0.327–0.764; *p* = 0.001), and 58.9% for influenza-related ICU admission (HR, 0.411; 95% CI, 0.146–1.161; *p* = 0.093). Although VE point estimates were numerically higher during higher-concordance seasons, there was no statistically significant evidence of heterogeneity in VE for medically attended influenza according to season-level antigenic concordance (*p* for interaction = 0.209). Covariate balance remained excellent after re-estimation of the overlap weights in both the repeat-vaccination and antigenic-concordance analyses, with maximum absolute standardized mean differences effectively equal to zero and well below the prespecified 0.10 threshold. In the outcome-specificity sensitivity analysis restricted to ICD-10 J10-coded events, the protective associations remained evident. For J10-coded medically attended influenza, 3691 events were identified, and the HR was 0.311 (95% CI, 0.224–0.430), corresponding to a VE of 68.9% (95% CI, 57.0–77.6%; *p* < 0.001). For J10-coded influenza hospitalization, 3132 events were identified, and the HR was 0.345 (95% CI, 0.246–0.484), corresponding to a VE of 65.5% (95% CI, 51.6–75.4%; *p* < 0.001). These findings were consistent with the direction of the primary analyses. Taken together, the sensitivity analyses showed that the protective associations persisted across alternative vaccination-window definitions, winter-season restriction, exclusion of repeat vaccination, season-level antigenic-concordance classifications, and the more specific J10-coded outcome definition. Influenza VE may vary with time since vaccination, circulating viral subtype, and vaccine–virus antigenic concordance [40], while observational VE estimates remain susceptible to residual confounding, including confounding by indication and healthy-vaccinee bias [41,42]. Accordingly, these analyses were interpreted primarily as robustness assessments rather than as causal evaluations of waning, repeat-vaccination effects, or individual-level vaccine–virus matching.

### 3.6. Exploratory Product-Specific Analyses

Exploratory analyses were conducted according to manufacturer code and vaccine valency. Prior real-world studies among older adults have reported variation in relative effectiveness across influenza vaccine formulations [43,44]. Given the unidentified manufacturer codes, incomplete product information, sparse outcome events, and the absence of multiplicity adjustment, detailed product-specific estimates are provided in Supplementary Table S1 and should be interpreted as hypothesis-generating rather than as confirmatory or head-to-head comparative-effectiveness findings.

## 4. Discussion

In this large, multi-season retrospective cohort of adults aged ≥60 years in Yichang, influenza vaccination was associated with lower hazards of medically attended influenza, influenza hospitalization, and influenza-related ICU admission, with overall VE estimates of approximately 53–56%. Protective associations were consistently observed across the two winter seasons and remained evident across age, sex, and chronic-disease subgroups, although the observed difference by sex should be regarded as exploratory. The primary findings were further supported by sensitivity analyses addressing vaccination timing, restriction to winter influenza seasons, repeat vaccination history, and season-level vaccine–virus antigenic concordance. A key contribution of this study is the integrated evaluation of influenza VE across multiple seasons and a clinically ordered spectrum of outcomes, from medically attended disease to hospitalization and ICU admission, within a single large linked-record cohort. Overall, these findings are broadly consistent with recent Chinese evidence showing moderate influenza VE among older adults, while also illustrating that the magnitude and precision of protection may vary across seasons, circulating strains, clinical outcomes, and study designs [12,45].

Recent evidence from China generally supports a moderate protective effect of influenza vaccination in older adults, although reported estimates vary substantially across settings and study designs. A three-season study among adults aged ≥60 years in Zhejiang Province reported an overall VE of 47.2% against laboratory-confirmed influenza, with additional variation by viral subtype and age [12]. A recent systematic review and meta-analysis of studies from China estimated a pooled VE of 33% against laboratory-confirmed influenza, with higher effectiveness observed for A(H1N1) pdm09 and during periods of better vaccine–circulating strain match [8]. Our overall estimates of approximately 53–56% are therefore somewhat higher than these pooled estimates but remain within the range reported in Chinese real-world studies. Direct numerical comparisons should nevertheless be made cautiously because VE estimates depend on outcome definition, circulating subtype, vaccine–virus match, population characteristics, healthcare-seeking patterns, and analytical design [5,8,12,34,45]. The present findings extend this evidence by demonstrating protective associations across both medically attended influenza and progressively more severe healthcare outcomes within a unified multi-season real-world framework.

Season-specific analyses further supported the consistency of the protective associations across the two winter seasons. Protective associations for medically attended influenza and influenza hospitalization were observed during both the 2023 and 2024 winter seasons, suggesting that the pooled estimates were not driven by a single influenza season. The antigenic-concordance sensitivity analysis provided additional epidemiological context to this seasonal variation. VE point estimates were numerically higher during the two higher-concordance periods than during the lower-concordance 2023 winter season across all three outcomes. This pattern is biologically plausible because influenza VE is known to vary according to circulating viral subtype and the degree of antigenic match between vaccine and circulating viruses [5,8,46,47]. However, the vaccination-by-concordance interaction for medically attended influenza was not statistically significant (*p* for interaction = 0.209), and concordance was assigned at the season rather than individual level. The findings should therefore be interpreted as contextual evidence consistent with seasonal heterogeneity rather than as evidence that vaccine–virus concordance causally modified individual-level VE. The larger point estimates observed during the 2024 summer season should likewise be interpreted cautiously because of the smaller number of events and wider confidence intervals, whereas the non-estimable estimates in the 2023 and 2025 summer seasons reflected sparse-data separation rather than complete vaccine protection. The predefined May–July and October–March follow-up windows were intended to capture recurrent periods of elevated summer and winter–spring influenza activity rather than to imply an absence of influenza circulation outside these intervals. Previous surveillance in Yichang has demonstrated complex annual, semiannual, and year-round influenza circulation patterns, including both summer and winter–spring epidemics, while longer-term surveillance in Hubei Province has shown a predominant winter–spring peak with a smaller summer increase [15,16]. Vaccination and healthcare records from August and September were retained and could contribute to subsequent-season exposure classification and baseline covariate assessment when they fell within the relevant pre-index windows.

The subgroup and sensitivity analyses provide additional support for the robustness of the primary findings. There was no statistical evidence of effect modification by age or chronic disease burden, indicating that the protective association remained broadly detectable across older-adult risk groups. The higher VE observed among women than among men was accompanied by a nominally significant interaction, although previous studies have also reported sex-related differences in influenza vaccine responses [35,36]. Given the borderline interaction *p*-value and the multiple subgroup comparisons performed, this finding should remain exploratory. Repeat vaccination within the preceding 365 days was uncommon, occurring in only 1.42% of vaccinated person-seasons, and exclusion of these observations followed by complete re-estimation of season-specific propensity scores and overlap weights did not materially alter the protective associations. This finding is consistent with previous evidence from older adults in China indicating that vaccination in consecutive seasons does not necessarily diminish the protection associated with current-season vaccination [11]. The primary 14–365-day exposure definition included a substantial proportion of person-seasons in which vaccination had occurred more than 180 days before the season-specific index date, particularly during the winter seasons. This raises the possibility that the primary definition captured vaccination from an earlier vaccination cycle in some person-seasons. However, the persistence of protective associations under progressively more restrictive 14–270-day, 14–180-day, and 14–90-day exposure definitions indicates that the observed associations were not dependent on the inclusion of more remote vaccination. Taken together, the restricted exposure-window analyses, winter-season restriction, repeat-vaccination exclusion, and antigenic-concordance analyses reduce the likelihood that the primary findings were attributable to any single exposure definition, season selection, or small group of repeatedly vaccinated individuals. Notably, VE against medically attended influenza was remarkably stable across the three restricted exposure windows (77.8–79.6%), whereas hospitalization VE point estimates were highest under the most restrictive 14–90-day definition. Although this pattern is broadly compatible with stronger protection following more recent vaccination, the overlapping exposure definitions and re-estimation of propensity-score weights under each specification preclude interpreting these comparisons as a formal analysis of within-season waning.

Scaled Schoenfeld-residual tests indicated non-proportionality of the vaccination effect for medically attended influenza and influenza hospitalization, whereas the proportional-hazards assumption was not rejected for influenza-related ICU admission. Accordingly, extended Cox models were used to characterize the vaccination associations for the first two outcomes over follow-up. The time-specific estimates showed stronger protective associations during the first 90 days and attenuated but persistent protective associations thereafter. Because the 90-day partition was defined according to follow-up time from the season-specific index date rather than time since vaccination, this pattern should not be interpreted as direct evidence of the biological waning of vaccine protection. The single Cox HRs for medically attended influenza and influenza hospitalization should therefore be viewed as summary associations over the observed follow-up, while the ≤90-day and >90-day estimates provide a more appropriate characterization of the time-varying associations.

This study has several strengths. First, the large population-based, multi-season cohort linked individual-level immunization and healthcare records, enabling the evaluation of influenza vaccine effectiveness across more than 1.1 million person-seasons. Second, three clinically relevant outcomes of increasing severity—medically attended influenza, influenza hospitalization, and influenza-related ICU admission—were evaluated within a common analytical framework. Third, season-specific propensity-score estimation and overlap weighting substantially improved balance in the measured baseline characteristics, while multiple sensitivity analyses addressing vaccination timing, restriction to winter seasons, repeat vaccination, and season-level antigenic concordance allowed the robustness of the primary findings to be examined under alternative analytical specifications. Finally, the multi-season design enabled an assessment of the consistency of the observed associations across different epidemiological and virological contexts.

Several limitations should also be acknowledged. Vaccinated and unvaccinated participants nevertheless differed substantially before weighting, particularly in comorbidity burden, healthcare utilization, and prior vaccination history, illustrating the potential for confounding in real-world influenza VE studies. In the present cohort, vaccinated participants had a greater burden of chronic disease and substantially higher healthcare utilization than unvaccinated participants, a pattern more consistent with confounding by indication and greater healthcare engagement than with a purely healthy-vaccinee effect. Similar baseline differences have been reported in large observational cohorts of older adults, in which vaccinated individuals often differ systematically from unvaccinated individuals in health status and healthcare use [48,49]. Such confounding by indication could bias crude estimates toward lower apparent vaccine effectiveness if individuals at greater baseline risk of influenza-related outcomes were preferentially vaccinated. Conversely, unmeasured differences in preventive-health behavior, functional status, frailty, or healthcare-seeking patterns could produce residual healthy-vaccinee or healthcare-engagement bias in the opposite direction. Overlap weighting achieved good balance in the measured baseline covariates, including near-exact balance after the re-estimation of weights in the repeat-vaccination and antigenic-concordance sensitivity analyses. The similarity of estimates from models with and without additional outcome-model covariate adjustment further suggests that the primary associations were not materially dependent on this modeling choice. Nevertheless, propensity-score weighting and sensitivity analyses cannot eliminate residual or unmeasured confounding. Accordingly, both residual confounding by indication and healthy-vaccinee-related bias should be considered when interpreting the observed associations [41,42,50]. Despite the low proportion of vaccinated person-seasons, propensity-score diagnostics demonstrated empirical common support across all five influenza seasons, with fewer than 1% of observations in either exposure group falling outside the common-support region in every season. These observations were retained in the weighted analyses, with overlap weighting limiting their influence through reduced weights rather than through explicit trimming. In addition, because overlap weighting targets the average treatment effect in the overlap population, the estimated associations primarily pertain to older adults with a meaningful probability of either receiving or not receiving an influenza vaccination and should not be assumed to apply uniformly to the entire older population in Yichang. Vaccination exposure classification also warrants caution. Although individual vaccination dates were available, lot-specific antigenic composition was unavailable for all vaccination records; therefore, exact correspondence between each administered vaccine and the seasonal formulation intended for the analyzed influenza period could not be verified directly. Importantly, the observed protective associations were not dependent on the inclusion of these more remote vaccinations. When the exposure window was restricted to 14–180 days before the index date, protective associations remained evident for medically attended influenza and influenza hospitalization, while the estimate for influenza-related ICU admission remained protective in direction but was less precise. Nevertheless, the restricted-window analysis addresses uncertainty related to vaccination timing rather than vaccine-formulation correspondence and therefore cannot establish that every recorded vaccination corresponded to the formulation intended for the subsequent influenza period.

In the primary analysis, censoring at subsequent influenza vaccination could have been informative because vaccination timing may have been related to evolving health status, healthcare utilization, or perceived infection risk. Baseline comparisons showed meaningful differences between subsequently vaccinated/censored and persistently unvaccinated person-seasons in several measured characteristics, particularly healthcare utilization, chronic respiratory disease, chronic disease burden, and prior receipt of non-influenza vaccines. However, vaccination-related censoring affected only 0.70% of initially unvaccinated person-seasons, and the time-varying exposure analysis yielded protective associations across all three outcomes. These findings reduce, although do not eliminate, concern that informative censoring materially influenced the primary estimates. However, the time-varying exposure analysis adjusted only for baseline covariates and did not incorporate time-updated health status, healthcare utilization, or other evolving factors during follow-up. Consequently, time-dependent confounding cannot be excluded if changes in these factors influenced both subsequent vaccination and the risk of influenza-related outcomes. The time-varying analysis should therefore be interpreted as addressing exposure reclassification and vaccination-related censoring rather than as fully accounting for time-dependent confounding.

Outcomes were identified from routinely collected healthcare records rather than systematic laboratory confirmation for every episode, and some degree of outcome misclassification is therefore possible. However, the protective associations persisted when the analysis was restricted to the more specific ICD-10 J10-coded outcome definition, reducing concern that the primary findings were driven solely by broader influenza diagnostic coding. Nevertheless, systematic laboratory confirmation was unavailable, and diagnosis-position information was not available in the analytic dataset; residual outcome misclassification therefore cannot be excluded. The unusually high ratio of influenza hospitalization to medically attended influenza events represents a major limitation of outcome ascertainment. Differential capture or coding of inpatient and outpatient influenza is one possible explanation, but this cannot be verified using the available data. Consequently, the observed ratio may reflect features of administrative-data ascertainment rather than the underlying probability of hospitalization among influenza infections, and the hospitalization findings should therefore be interpreted cautiously. Individual-level mortality data and dates of death were also unavailable in the linked data sources; consequently, non-influenza death could not be explicitly modeled as a competing event. This limitation may be particularly relevant when interpreting hospitalization and ICU outcomes in this older population. Season-specific estimates were imprecise when vaccinated outcome events were sparse, particularly during summer seasons. The exploratory product-specific analyses were additionally limited by unidentified manufacturer codes, incomplete manufacturer or valency information, small numbers of vaccinated outcome events, and the absence of multiplicity adjustment. Accordingly, these analyses have limited clinical interpretability and should be regarded as hypothesis-generating rather than as confirmatory evidence of differences between vaccine products. Antigenic concordance was assigned at the influenza-season level using surveillance data rather than individual-level viral typing or antigenic characterization. Accordingly, this analysis cannot determine whether individual breakthrough infections were caused by vaccine-matched or mismatched viruses, and only three study periods contributed to the concordance comparison. Finally, the study population was drawn from older adults represented in the Yichang healthcare and immunization data systems and required evidence of healthcare contact during the 365-day baseline period. Generalizability to older adults with little or no healthcare contact, or to populations in other regions of China, may therefore depend on local epidemiology, vaccination practices, healthcare-seeking behavior, and healthcare access.

The relatively low proportion of vaccinated person-seasons observed in this study should be interpreted in the context of the local vaccination environment. During the study period, influenza vaccination for older adults in Yichang was primarily voluntary and self-paid rather than being provided through a routine publicly funded program [51]. This policy context may have contributed to the low uptake observed in the analytic cohort. Beyond financing policy, previous studies in China have identified limited vaccine awareness, concerns regarding vaccine safety or perceived necessity, and insufficient healthcare-worker recommendation as additional contributors to low influenza vaccination uptake among older adults [52]. Importantly, the 2.85% figure represents the proportion of vaccinated person-seasons within the analytic cohort and should not be interpreted as the annual population-level vaccination coverage among all older adults in Yichang. The vaccination context in Yichang may therefore differ from settings with substantially higher uptake and broader public financing of influenza vaccination. Accordingly, the generalizability of the present estimates to settings with substantially different vaccination policies and coverage levels should be considered with caution.

Despite these limitations, influenza vaccination coverage among Chinese older adults remains low; a recent meta-analysis estimated pooled coverage of 17%, falling to 7% in areas without free-vaccination policies [8]. The WHO continues to identify older adults and people with underlying conditions as priority groups for seasonal influenza vaccination [19]. Recent economic evidence from China further suggests that influenza vaccination can be cost-effective and highlights the importance of high-quality local effectiveness and disease-burden data for informing vaccination policy [53]. Taken together, the present findings support continued efforts to improve seasonal influenza vaccination uptake among older adults while emphasizing the importance of evaluating VE across multiple seasons, clinical outcomes, vaccination histories, and changing virological contexts.

## 5. Conclusions

In this large, multi-season retrospective cohort of adults aged ≥60 years in Yichang, recorded influenza vaccination was associated with lower hazards of medically attended influenza, influenza hospitalization, and influenza-related ICU admission. Protective associations were consistently observed across the major winter influenza seasons and remained evident across prespecified older-adult subgroups. Protective associations also persisted across alternative vaccination windows, restriction to winter seasons, exclusion of repeat vaccination within the preceding 365 days, and analyses incorporating season-level vaccine–virus antigenic concordance. Although VE point estimates were numerically higher during periods of greater antigenic concordance, this difference was not statistically supported and should be interpreted cautiously. Given the observational design, residual confounding, outcome misclassification, and the overlap-population estimand, the findings should be interpreted as associations rather than definitive causal effects applicable to all older adults. Overall, these findings provide supportive real-world evidence that influenza vaccination is associated with lower influenza-related morbidity among older adults in Yichang, while warranting cautious generalization beyond the overlap population represented by the study.

## Figures and Tables

**Table 1:** Baseline characteristics of vaccinated and unvaccinated person-seasons before and after overlap weighting.

Characteristic	Unweighted		Absolute SMD	Overlap-Weighted		Absolute SMD
	Unvaccinated	Vaccinated		Unvaccinated	Vaccinated	
Person-seasons	1,075,346	31,540		27,069.8	27,051.9	
Age, years, mean (SD)	70.51 (7.48)	71.76 (7.18)	0.169	71.73 (7.20)	71.75 (7.20)	0.003
Sex, *n* (%)			0.182			0.005
Male	450,809 (41.9)	12,953 (41.1)		11,021.2 (40.7)	10,964.5 (40.5)	
Female	473,134 (44.0)	15,819 (50.2)		13,510.3 (49.9)	13,563.2 (50.1)	
Unknown	151,403 (14.1)	2768 (8.8)		2538.4 (9.4)	2524.2 (9.3)	
Hypertension, *n* (%)	274,520 (25.5)	11,611 (36.8)	0.245	9888.5 (36.5)	9822.9 (36.3)	0.005
Diabetes mellitus, *n* (%)	126,294 (11.7)	5170 (16.4)	0.134	4427.2 (16.4)	4402.3 (16.3)	0.002
Chronic respiratory disease, *n* (%)	134,437 (12.5)	7169 (22.7)	0.271	5850.7 (21.6)	5812.4 (21.5)	0.003
Ischemic heart disease, *n* (%)	141,213 (13.1)	6920 (21.9)	0.233	5786.7 (21.4)	5759.6 (21.3)	0.002
Heart failure, *n* (%)	51,078 (4.7)	2485 (7.9)	0.129	2094.0 (7.7)	2079.1 (7.7)	0.002
Cerebrovascular disease, *n* (%)	117,500 (10.9)	4973 (15.8)	0.143	4259.6 (15.7)	4222.3 (15.6)	0.004
Chronic kidney disease, *n* (%)	23,525 (2.2)	1084 (3.4)	0.076	927.7 (3.4)	917.8 (3.4)	0.002
Cancer, *n* (%)	41,838 (3.9)	1625 (5.2)	0.061	1346.3 (5.0)	1379.8 (5.1)	0.006
≥2 chronic conditions, *n* (%)	258,609 (24.0)	12,079 (38.3)	0.311	10,228.7 (37.8)	10,086.9 (37.3)	0.010
Outpatient contact days, mean (SD)	4.97 (7.09)	8.35 (8.81)	0.423	8.56 (10.38)	8.11 (8.63)	0.048
Inpatient contact days, mean (SD)	1.03 (2.48)	1.60 (2.95)	0.210	1.62 (3.17)	1.54 (2.90)	0.026
Total unique healthcare contact days, mean (SD)	5.79 (7.77)	9.60 (9.50)	0.439	9.83 (11.05)	9.31 (9.31)	0.050
Prior influenza diagnosis, *n* (%)	3839 (0.4)	209 (0.7)	0.043	155.5 (0.6)	161.6 (0.6)	0.003
Any non-influenza vaccination, *n* (%)	9431 (0.9)	4769 (15.1)	0.544	1251.0 (4.6)	1259.5 (4.7)	0.002
Pneumococcal vaccination, *n* (%)	703 (0.1)	4019 (12.7)	0.536	589.6 (2.2)	589.4 (2.2)	<0.001

**Note**: Values are presented as *n* (%) unless otherwise indicated. Weighted values are sums of overlap weights and represent pseudo-population totals rather than numbers of unique individuals or effective sample sizes; therefore, they may be non-integer. Propensity scores and overlap weights were estimated separately within each influenza season. SMDs are presented as absolute values; an absolute SMD < 0.10 was considered indicative of adequate covariate balance. For categorical variables with more than two levels, the SMD shown on the first category row represents the overall standardized difference for that variable. “Unknown” sex denotes records coded as unknown in the source database and was retained as a separate category in the propensity-score model. The ≥2 chronic conditions row was derived by combining the 2-condition and ≥3-condition categories. “Any non-influenza vaccination” refers to receipt of at least one vaccine other than influenza vaccine during the baseline period; pneumococcal vaccination is additionally reported separately because of its clinical relevance in older adults. Total unique healthcare contact days were calculated as unique calendar days with any outpatient or inpatient encounter and therefore need not equal the sum of outpatient and inpatient contact days. SMD, standardized mean difference. Complete covariate balance is shown in Table A2.

**Table 2:** Season-specific propensity-score overlap and effective-sample-size diagnostics.

Season	Unvaccinated, *n*	Vaccinated, *n*	Common-Support Range	Unvaccinated Outside Support, %	Vaccinated Outside Support, %	Unvaccinated ESS	Vaccinated ESS	Overall ESS
2023 Summer	150,404	5123	0.0036–0.9723	0.33	0.04	72,210	4622	17,448
2023 Winter	238,713	5943	0.0031–0.9515	0.82	0.03	85,705	5442	20,459
2024 Summer	183,859	6780	0.0032–0.9732	0.17	0.00	90,014	6071	22,756
2024 Winter	284,505	7414	0.0027–0.9591	0.24	0.20	125,502	6702	25,477
2025 Summer	217,865	6280	0.0030–0.9741	0.07	0.03	119,093	5771	21,982

**Note:** Common support was defined as the intersection of the observed propensity-score ranges among the vaccinated and unvaccinated person-seasons within each influenza season. Percentages outside common support were calculated separately within each exposure group. ESS was calculated from the overlap weights as $ESS=\frac{{\left(\sum w_{i}\right)}^{2}}{\sum w_{i}}$. Propensity scores and overlap weights were estimated separately within each influenza season. The common-support assessment was diagnostic only. Observations outside the empirical common-support interval were retained in the primary overlap-weighted analyses and were not excluded by propensity-score trimming.

**Table 3:** Primary estimates of influenza vaccine effectiveness.

Outcome	Estimate/Follow-Up Period	Events, *n*	Schoenfeld-Residual Test *p*-Value	HR (95% CI)	VE, % (95% CI)	*p*-Value
Medically attended influenza diagnosis	Summary Cox estimate	5463	<0.001	0.443 (0.347–0.566)	55.7 (43.4–65.3)	<0.001
	≤90 days	—	—	0.101 (0.045–0.225)	—	—
	>90 days	—	—	0.469 (0.355–0.620)	—	—
Hospitalization with an influenza diagnosis	Summary Cox estimate	3946	<0.001	0.456 (0.343–0.606)	54.4 (39.4–65.7)	<0.001
	≤90 days	—	—	0.076 (0.025–0.237)	—	—
	>90 days	—	—	0.490 (0.358–0.670)	—	—
Influenza-related ICU admission	Summary Cox estimate	865	0.159	0.473 (0.261–0.858)	52.7 (14.2–73.9)	0.014

**Note:** Summary hazard ratios were estimated using overlap-weighted Cox models with additional adjustment for baseline covariates, stratification by influenza season, and robust variance estimation clustered at the individual level. The proportional hazards assumption for the vaccination effect was assessed using scaled Schoenfeld residuals. Because treatment-specific non-proportionality was detected for medically attended influenza and influenza hospitalization, extended-Cox estimates are additionally presented separately for ≤90 and >90 days of follow-up. For these two outcomes, the summary Cox estimates should be interpreted as overall associations across the observed follow-up rather than as constant hazard ratios. The proportional hazards assumption was not rejected for influenza-related ICU admission. HR, hazard ratio; CI, confidence interval; VE, vaccine effectiveness; ICU, intensive care unit.

**Table 4:** Overlap-weighted event risks according to influenza vaccination status.

Outcome	Vaccination Status	Observed Events, *n*	Weighted Person-Seasons	Weighted Event Risk per 1000 Person-Seasons
Medically attended influenza	Unvaccinated	5398	27,069.8	5.58
	Vaccinated	65	27,051.9	1.92
Influenza hospitalization	Unvaccinated	3898	27,069.8	4.07
	Vaccinated	48	27,051.9	1.47
Influenza-related ICU admission	Unvaccinated	854	27,069.8	1.09
	Vaccinated	11	27,051.9	0.33

Note: Event counts represent unweighted observed events, whereas event risks and weighted person-season totals are overlap-weighted estimates for the overlap population. Weighted totals represent pseudo-population quantities and should not be interpreted as actual numbers of individuals or events.

**Table 5:** Season-specific estimates of influenza vaccine effectiveness.

Influenza Season	Outcome	*N*	Events, *n*	HR (95% CI)	VE, %	*p*-Value
2023 Summer	Medically attended influenza	155,527	154	Not estimable	Not estimable	—
	Influenza hospitalization	155,527	79	Not estimable	Not estimable	—
	Influenza-related ICU admission	155,527	17	Not estimable	Not estimable	—
2023 Winter	Medically attended influenza	244,656	2103	0.551 (0.382–0.795)	44.9	0.001
	Influenza hospitalization	244,656	1532	0.601 (0.398–0.907)	39.9	0.015
	Influenza-related ICU admission	244,656	273	0.588 (0.219–1.577)	41.2	0.291
2024 Summer	Medically attended influenza	190,639	428	0.127 (0.032–0.510)	87.3	0.004
	Influenza hospitalization	190,639	254	0.215 (0.054–0.865)	78.5	0.030
	Influenza-related ICU admission	190,639	76	0.361 (0.050–2.600)	63.9	0.312
2024 Winter	Medically attended influenza	291,919	2657	0.491 (0.350–0.689)	50.9	<0.001
	Influenza hospitalization	291,919	2032	0.434 (0.288–0.655)	56.6	<0.001
	Influenza-related ICU admission	291,919	493	0.468 (0.209–1.047)	53.2	0.065
2025 Summer	Medically attended influenza	224,145	121	Not estimable	Not estimable	—
	Influenza hospitalization	224,145	49	Not estimable	Not estimable	—
	Influenza-related ICU admission	224,145	6	Not estimable	Not estimable	—

**Note:** Hazard ratios were estimated using overlap-weighted Cox proportional hazards models fitted separately within each influenza season, with additional adjustment for baseline covariates and robust variance estimation clustered at the individual level. Vaccine effectiveness was calculated as *VE* = (1 − *HR*) × 100%. Estimates were reported as not estimable when sparse events in the vaccinated group resulted in model separation. HR, hazard ratio; CI, confidence interval; VE, vaccine effectiveness; ICU, intensive care unit.

**Table 6:** Influenza vaccine effectiveness for medically attended influenza in prespecified subgroups.

Subgroup	Level	HR (95% CI)	VE, % (95% CI)	*p*-Value	*p* for Interaction
Age group	60–69 years	0.396 (0.263–0.596)	60.4 (40.4–73.7)	<0.001	0.564
	70–79 years	0.317 (0.213–0.472)	68.3 (52.8–78.7)	<0.001	
	≥80 years	0.270 (0.134–0.543)	73.0 (45.7–86.6)	<0.001	
Sex	Female	0.200 (0.122–0.329)	80.0 (67.1–87.8)	<0.001	0.045
	Male	0.445 (0.321–0.618)	55.5 (38.2–67.9)	<0.001	
Chronic disease burden	0–1 conditions	0.282 (0.187–0.424)	71.8 (57.6–81.3)	<0.001	0.249
	≥2 conditions	0.390 (0.276–0.552)	61.0 (44.8–72.4)	<0.001	

**Note:** Subgroup estimates were derived from overlap-weighted Cox proportional hazards models for medically attended influenza, with additional adjustment for baseline covariates, stratification by influenza season, and robust variance estimation clustered at the individual level. Effect modification was assessed using vaccination-by-subgroup interaction terms. The interaction *p*-value is shown once for each subgroup factor. VE was calculated as *VE* = (1 − *HR*) × 100%. HR, hazard ratio; CI, confidence interval; VE, vaccine effectiveness.

**Table 7:** Sensitivity analyses of influenza vaccine effectiveness under alternative analytical specifications.

Analysis	Outcome	*N*	Events, *n*	HR (95% CI)	VE, % (95% CI)	*p*-Value
Time-varying vaccination exposure, 14-day lag	Medically attended influenza	1,106,886	5495	0.371 (0.301–0.459)	62.9 (54.1–69.9)	<0.001
	Influenza hospitalization	1,106,886	3968	0.366 (0.286–0.469)	63.4 (53.1–71.4)	<0.001
	Influenza-related ICU admission	1,106,886	868	0.276 (0.155–0.492)	72.4 (50.8–84.5)	<0.001
Restricted vaccination window, 14–90 days	Medically attended influenza	1,078,698	5407	0.222 (0.094–0.524)	77.8 (47.6–90.6)	<0.001
	Influenza hospitalization	1,078,698	3900	0.079 (0.015–0.409)	92.1 (59.1–98.5)	0.002
	Influenza-related ICU admission	1,078,698	855	0.251 (0.035–1.811)	74.9 (−81.1–96.5)	0.171
Restricted vaccination window, 14–180 days	Medically attended influenza	1,085,335	5411	0.216 (0.109–0.428)	78.4 (57.2–89.1)	<0.001
	Influenza hospitalization	1,085,335	3904	0.169 (0.068–0.425)	83.1 (57.5–93.2)	<0.001
	Influenza-related ICU admission	1,085,335	857	0.418 (0.132–1.325)	58.2 (−32.5–86.8)	0.138
Restricted vaccination window, 14–270 days	Medically attended influenza	1,097,318	5420	0.204 (0.123–0.338)	79.6 (66.2–87.7)	<0.001
	Influenza hospitalization	1,097,318	3912	0.216 (0.120–0.391)	78.4 (60.9–88.0)	<0.001
	Influenza-related ICU admission	1,097,318	860	0.349 (0.142–0.860)	65.1 (14.0–85.8)	0.022
Winter seasons only	Medically attended influenza	536,575	4760	0.393 (0.301–0.514)	60.7 (48.6–69.9)	<0.001
	Influenza hospitalization	536,575	3564	0.396 (0.291–0.539)	60.4 (46.1–70.9)	<0.001
	Influenza-related ICU admission	536,575	766	0.344 (0.179–0.662)	65.6 (33.8–82.1)	0.001
Excluding repeat vaccination within 365 days	Medically attended influenza	1,106,438	5461	0.339 (0.260–0.442)	66.1 (55.8–74.0)	<0.001
	Influenza hospitalization	1,106,438	3945	0.357 (0.264–0.484)	64.3 (51.6–73.6)	<0.001
	Influenza-related ICU admission	1,106,438	865	0.298 (0.156–0.568)	70.2 (43.2–84.4)	<0.001
Higher antigenic concordance seasons	Medically attended influenza	482,558	3085	0.303 (0.212–0.434)	69.7 (56.6–78.8)	<0.001
	Influenza hospitalization	482,558	2286	0.283 (0.183–0.436)	71.7 (56.4–81.7)	<0.001
	Influenza-related ICU admission	482,558	569	0.258 (0.110–0.609)	74.2 (39.1–89.0)	0.002
Lower antigenic concordance season	Medically attended influenza	244,656	2103	0.426 (0.289–0.629)	57.4 (37.1–71.1)	<0.001
	Influenza hospitalization	244,656	1532	0.500 (0.327–0.764)	50.0 (23.6–67.3)	0.001
	Influenza-related ICU admission	244,656	273	0.411 (0.146–1.161)	58.9 (−16.1–85.4)	0.093
J10-restricted outcome definition	Medically attended influenza	1,106,886	3691	0.311 (0.224–0.430)	68.9 (57.0–77.6)	<0.001
	Influenza hospitalization	1,106,886	3132	0.345 (0.246–0.484)	65.5 (51.6–75.4)	<0.001

**Note:** Except for the time-varying vaccination analysis, sensitivity estimates were derived from overlap-weighted Cox proportional hazards models with additional adjustment for baseline covariates, stratification by influenza season where applicable, and robust variance estimation clustered at the individual level. In the time-varying vaccination analysis, initially unvaccinated participants contributed unvaccinated person-time until 14 days after subsequent vaccination and vaccinated person-time thereafter; the model was adjusted for the prespecified baseline covariates, stratified by influenza season, and used individual-level clustered robust variance estimates. For the alternative 14–90-day, 14–180-day, and 14–270-day vaccination-window analyses, the analysis excluding repeat vaccination within 365 days, and the antigenic-concordance analyses, propensity scores and overlap weights were re-estimated after applying the respective alternative exposure or sample definitions. Higher antigenic concordance included the 2024 summer and 2024 winter seasons, whereas lower antigenic concordance comprised the 2023 winter season; the virological evidence underlying these classifications is summarized in Table A3. Antigenic concordance was defined at the influenza-season level and should not be interpreted as individual-level vaccine–virus matching. For the outcome-specificity sensitivity analysis, influenza outcomes were restricted to ICD-10 J10-coded events, representing influenza due to an identified influenza virus; these records should not be interpreted as systematic laboratory confirmation of every event. In the time-varying vaccination analysis, *N* refers to the number of original person-seasons rather than the expanded start–stop intervals used for model fitting. VE was calculated as *VE* = (1 − *HR*) × 100%. Negative lower confidence limits for VE indicate that the corresponding HR confidence interval crossed 1. HR, hazard ratio; CI, confidence interval; VE, vaccine effectiveness; ICU, intensive care unit.

## Data Availability

The individual-level data analyzed in this study were obtained from the Yichang Center for Disease Control and Prevention (Yichang CDC) and contain confidential health information. Owing to privacy considerations, institutional data-governance requirements, and restrictions under the applicable data-use agreements, the data cannot be deposited in a public repository or shared publicly. Record linkage and anonymization were completed by Yichang CDC before investigators accessed the analytical dataset. All analyses were conducted within a secure computing environment authorized by Yichang CDC. Access to the data is controlled by Yichang CDC and may be considered only upon reasonable request to the corresponding author and subject to approval by Yichang CDC and the relevant ethics and data-governance authorities.

## Supplementary Materials

The following supporting information can be downloaded at: https://www.mdpi.com/article/10.3390/vaccines14100858/s1, Table S1: Exploratory Product-Specific Estimates for Medically Attended Influenza; STROBE Checklist: STROBE checklist for cohort studies.

## Abbreviations

The following abbreviations are used in this manuscript:

CDC	Center for Disease Control and Prevention
CI	Confidence interval
HR	Hazard ratio
ICD-10	International Classification of Diseases, Tenth Revision
ICU	Intensive care unit
NSFC	National Natural Science Foundation of China
PS	Propensity score
SD	Standard deviation
SMD	Standardized mean difference
STROBE	Strengthening the Reporting of Observational Studies in Epidemiology
VE	Vaccine effectiveness
ESS	Effective Sample Size
WHO	World Health Organization

## Appendix A

### Appendix A.1. ICD-10 Code Definitions

To enhance transparency and reproducibility of the case definitions used in this study, Table A1 summarizes the prespecified ICD-10 code definitions applied to identify influenza-related outcomes, baseline comorbidities, and immunocompromising conditions used for cohort exclusion.

**Table A1:** ICD-10 code definitions for influenza outcomes, baseline comorbidities, and immunocompromising exclusion conditions.

Category	Condition/Definition	ICD-10 Code(s)
Influenza outcomes	Medically attended influenza	J09–J11
	Influenza hospitalization	J09–J11
	Influenza-related ICU admission	J09–J11, with ICU admission occurring from 1 day before through 14 days after the first qualifying influenza diagnosis *
Baseline comorbidities	Hypertension	I10–I15
	Diabetes mellitus	E10–E14
	Chronic respiratory disease	J40–J47
	Ischemic heart disease	I20–I25
	Heart failure	I50
	Cerebrovascular disease	I60–I69
	Chronic kidney disease	N18
	Cancer	C00–C99
	Dementia	F00–F03; G30
	Chronic liver disease	K70–K76
	Tuberculosis	A15–A19
Immunocompromising exclusion conditions	Immunocompromising conditions	D80–D89; B20–B24; Z94; Z48.2

**Note:** ICD-10, International Classification of Diseases, Tenth Revision; ICU, intensive care unit. Medically attended influenza was identified from qualifying outpatient or inpatient diagnoses coded J09–J11. Influenza hospitalization required a qualifying J09–J11 diagnosis recorded during an inpatient encounter. * Influenza-related ICU admission was defined by temporal linkage rather than by a separate ICD-10 diagnosis code; an ICU encounter was considered influenza-related when it occurred from 1 day before through 14 days after the first qualifying influenza diagnosis.

### Appendix A.2. Complete Covariate Balance Table

Table A2 presents the complete covariate balance diagnostics before and after overlap weighting. While Table 1 in the main manuscript summarizes selected baseline characteristics, Table A2 reports the full set of covariates included in the propensity score model, together with their absolute SMDs. Before weighting, several characteristics differed appreciably between vaccinated and unvaccinated person-seasons. As shown in Table A2, overlap weighting substantially improved balance across all measured covariates, with all absolute SMDs below 0.10, indicating adequate covariate balance between the two exposure groups.

**Table A2:** Complete covariate balance of vaccinated and unvaccinated person-seasons before and after overlap weighting.

Characteristic	Level	Unweighted		Absolute SMD	Overlap-Weighted		Absolute SMD
		Unvaccinated	Vaccinated		Unvaccinated	Vaccinated	
Person-seasons		1,075,346	31,540		27,069.82	27,051.91	
Age, years, mean (SD)		70.51 (7.48)	71.76 (7.18)	0.169	71.73 (7.20)	71.75 (7.20)	0.003
Sex, *n* (%)	Male	450,809 (41.9)	12,953 (41.1)	0.182	11,021.2 (40.7)	10,964.5 (40.5)	0.005
	Female	473,134 (44.0)	15,819 (50.2)		13,510.3 (49.9)	13,563.2 (50.1)	
	Unknown	151,403 (14.1)	2768 (8.8)		2538.4 (9.4)	2524.2 (9.3)	
Hypertension, *n* (%)	No	800,826 (74.5)	19,929 (63.2)	0.245	17,181.3 (63.5)	17,229.1 (63.7)	0.005
	Yes	274,520 (25.5)	11,611 (36.8)		9888.5 (36.5)	9822.9 (36.3)	
Diabetes mellitus, *n* (%)	No	949,052 (88.3)	26,370 (83.6)	0.134	22,642.6 (83.6)	22,649.6 (83.7)	0.002
	Yes	126,294 (11.7)	5170 (16.4)		4427.2 (16.4)	4402.3 (16.3)	
Chronic respiratory disease, *n* (%)	No	940,909 (87.5)	24,371 (77.3)	0.271	21,219.1 (78.4)	21,239.5 (78.5)	0.003
	Yes	134,437 (12.5)	7169 (22.7)		5850.7 (21.6)	5812.4 (21.5)	
Ischemic heart disease, *n* (%)	No	934,133 (86.9)	24,620 (78.1)	0.233	21,283.1 (78.6)	21,292.3 (78.7)	0.002
	Yes	141,213 (13.1)	6920 (21.9)		5786.7 (21.4)	5759.6 (21.3)	
Heart failure, *n* (%)	No	1,024,268 (95.3)	29,055 (92.1)	0.129	24,975.8 (92.3)	24,972.8 (92.3)	0.002
	Yes	51,078 (4.7)	2485 (7.9)		2094.0 (7.7)	2079.1 (7.7)	
Cerebrovascular disease, *n* (%)	No	957,846 (89.1)	26,567 (84.2)	0.143	22,810.3 (84.3)	22,829.6 (84.4)	0.004
	Yes	117,500 (10.9)	4973 (15.8)		4259.6 (15.7)	4222.3 (15.6)	
Chronic kidney disease, *n* (%)	No	1,051,821 (97.8)	30,456 (96.6)	0.076	26,142.2 (96.6)	26,134.1 (96.6)	0.002
	Yes	23,525 (2.2)	1084 (3.4)		927.7 (3.4)	917.8 (3.4)	
Cancer, *n* (%)	No	1,033,508 (96.1)	29,915 (94.8)	0.061	25,723.5 (95.0)	25,672.1 (94.9)	0.006
	Yes	41,838 (3.9)	1625 (5.2)		1346.3 (5.0)	1379.8 (5.1)	
Dementia, *n* (%)	No	1,071,409 (99.6)	31,337 (99.4)	0.039	26,890.2 (99.3)	26,877.1 (99.4)	0.002
	Yes	3937 (0.4)	203 (0.6)		179.6 (0.7)	174.8 (0.6)	
Chronic liver disease, *n* (%)	No	1,031,308 (95.9)	29,337 (93.0)	0.127	25,271.5 (93.4)	25,258.9 (93.4)	0.001
	Yes	44,038 (4.1)	2203 (7.0)		1798.4 (6.6)	1793.0 (6.6)	
Tuberculosis, *n* (%)	No	1,048,965 (97.5)	29,974 (95.0)	0.133	25,793.0 (95.3)	25,758.7 (95.2)	0.003
	Yes	26,381 (2.5)	1566 (5.0)		1276.9 (4.7)	1293.2 (4.8)	
Number of chronic conditions, *n* (%)	0	581,746 (54.1)	11,425 (36.2)	0.387	9920.3 (36.6)	10,073.4 (37.2)	0.014
	1	234,991 (21.9)	8036 (25.5)		6920.8 (25.6)	6891.6 (25.5)	
	2	127,680 (11.9)	5334 (16.9)		4597.2 (17.0)	4485.5 (16.6)	
	≥3	130,929 (12.2)	6745 (21.4)		5631.5 (20.8)	5601.4 (20.7)	
Outpatient contact days, mean (SD)		4.97 (7.09)	8.35 (8.81)	0.423	8.56 (10.38)	8.11 (8.63)	0.048
Inpatient contact days, mean (SD)		1.03 (2.48)	1.60 (2.95)	0.210	1.62 (3.17)	1.54 (2.90)	0.026
Total unique healthcare contact days, mean (SD)		5.79 (7.77)	9.60 (9.50)	0.439	9.83 (11.05)	9.31 (9.31)	0.05
Prior influenza diagnosis, *n* (%)	No	1,071,507 (99.6)	31,331 (99.3)	0.043	26,914.3 (99.4)	26,890.4 (99.4)	0.003
	Yes	3839 (0.4)	209 (0.7)		155.5 (0.6)	161.6 (0.6)	
Any non-influenza vaccination, *n* (%)	No	1,065,915 (99.1)	26,771 (84.9)	0.544	25,818.8 (95.4)	25,792.4 (95.3)	0.002
	Yes	9431 (0.9)	4769 (15.1)		1251.0 (4.6)	1259.5 (4.7)	
Pneumococcal vaccination, *n* (%)	No	1,074,643 (99.9)	27,521 (87.3)	0.536	26,480.3 (97.8)	26,462.6 (97.8)	<0.001
	Yes	703 (0.1)	4019 (12.7)		589.6 (2.2)	589.4 (2.2)	
Influenza season, *n* (%)	2023 summer	150,404 (14.0)	5123 (16.2)	0.150	4369.2 (16.1)	4349.0 (16.1)	0.002
	2023 winter	238,713 (22.2)	5943 (18.8)		5136.9 (19.0)	5139.1 (19.0)	
	2024 summer	183,859 (17.1)	6780 (21.5)		5696.4 (21.0)	5694.6 (21.1)	
	2024 winter	284,505 (26.5)	7414 (23.5)		6375.5 (23.6)	6368.2 (23.5)	
	2025 summer	217,865 (20.3)	6280 (19.9)		5491.8 (20.3)	5501.0 (20.3)	

**Note:** Values are presented as *n* (%) unless otherwise indicated. Weighted values are sums of overlap weights and represent pseudo-population totals rather than number of unique individuals or effective sample sizes; therefore, they may be non-integer. Propensity scores and overlap weights were estimated separately within each influenza season. SMDs are presented as absolute values; an absolute SMD < 0.10 was considered indicative of adequate covariate balance. For categorical variables, the SMD shown on the first category row is the overall standardized difference for that variable. “Unknown” sex denotes records coded as unknown in the source database and was retained as a separate category in the propensity-score model. “Any non-influenza vaccination” refers to receipt of at least one vaccine other than the influenza vaccine during the baseline period; pneumococcal vaccination is additionally reported separately because of its clinical relevance in older adults. Total unique healthcare contact days were calculated as unique calendar days with any outpatient or inpatient encounter and therefore need not equal the sum of outpatient and inpatient contact days. SMD, standardized mean difference.

### Appendix A.3. Season-Specific Covariate Balance Diagnostics

Figure A1 presents the season-specific covariate balance diagnostics before and after overlap weighting for each included influenza season. Before weighting, several baseline covariates showed appreciable imbalance between vaccinated and unvaccinated person-seasons, particularly measures of healthcare utilization, prior vaccination, age, and selected chronic conditions. As shown in Figure A1, overlap weighting substantially improved covariate balance in every season, with the weighted absolute standardized mean differences for all measured covariates falling below 0.10. These results support the adequacy of the season-specific propensity score models and indicate that good within-season comparability was achieved between the two exposure groups after weighting.

### Appendix A.4. Virological Context and Season-Level Antigenic Concordance Classification

To provide virological context for the season-level antigenic-concordance sensitivity analysis, information on predominant circulating influenza subtypes and antigenic characterization was obtained from contemporaneous surveillance reports of the Chinese National Influenza Center. Classification was based on the predominant circulating subtype during each study period and the proportion of antigenically characterized viruses reported as similar to the corresponding vaccine-like strain. The virological characteristics and resulting season-level concordance classifications are summarized in Table A3.

The 2023 winter season was classified as a lower-concordance period. A(H3N2) viruses predominated during this period, and 47.0% of antigenically characterized A(H3N2) viruses were reported as A/Darwin/9/2021 (H3N2)-like [54]. In contrast, A(H1N1) pdm09 viruses predominated during the 2024 summer and winter periods; 97.2% and 96.8% of characterized A(H1N1) pdm09 viruses, respectively, were reported as A/Victoria/4897/2022-like [55,56]. These two periods were therefore classified as higher-concordance seasons.

**Table A3:** Virological context and season-level vaccine–virus antigenic concordance used in the sensitivity analysis.

Study Period	Predominant Circulating Subtype	Relevant Vaccine-Like Strain	Antigenically Similar Viruses, %	Concordance Classification
2023 Winter	A (H3N2)	A/Darwin/9/2021 (H3N2)-like	47.0	Lower concordance
2024 Summer	A (H1N1) pdm09	A/Victoria/4897/2022 (H1N1) pdm09-like	97.2	Higher concordance
2024 Winter	A (H1N1) pdm09	A/Victoria/4897/2022 (H1N1) pdm09-like	96.8	Higher concordance

**Note:** Antigenic-concordance classification was defined at the influenza-season level using CNIC surveillance data and was intended to provide epidemiological context for the sensitivity analysis. The classification does not represent individual-level viral typing or direct vaccine–virus matching for individual influenza cases. The 2023 summer and 2025 summer study periods were not classified for this analysis because they were not included in the predefined concordance comparison.

### Appendix A.5. Post-Index Vaccination and Potential Informative Censoring

To further evaluate the potential for informative censoring arising from influenza vaccination during follow-up, we characterized person-seasons that were unvaccinated at the season-specific index date but subsequently received influenza vaccination. We quantified vaccination-related censoring within each influenza season and compared baseline characteristics between subsequently vaccinated/censored person-seasons and those that remained unvaccinated throughout follow-up. The corresponding results are presented in Table A4.

**Table A4:** Vaccination-related censoring and baseline characteristics of initially unvaccinated person-seasons.

Vaccination-Related Censoring by Influenza Season			
**Influenza Season**	**Unvaccinated at Index, *n***	**Subsequently Vaccinated/Censored, *n***	**Censored, %**
2023 Summer	150,404	41	0.03
2023 Winter	238,713	4370	1.83
2024 Summer	183,859	57	0.03
2024 Winter	284,505	2972	1.04
2025 Summer	217,865	45	0.02
Overall	1,075,346	7485	0.70
**Baseline Characteristics of Person-Seasons Remaining Unvaccinated Versus Subsequently Vaccinated/Censored**			
**Characteristic**	**Unvaccinated** **(*n* = 1,067,861)**	**Subsequently Vaccinated/Censored** **(*n* = 7485)**	**Absolute SMD**
Age, years	70.51 (7.48)	70.83 (7.28)	0.044
Outpatient contact days	4.96 (7.08)	6.29 (8.06)	0.176
Inpatient contact days	1.03 (2.48)	1.45 (2.68)	0.161
Total healthcare contact days	5.78 (7.76)	7.44 (8.78)	0.200
Number of chronic conditions	0.91 (1.31)	1.18 (1.47)	0.190
Hypertension	272,268 (25.5)	2252 (30.1)	0.103
Diabetes mellitus	125,327 (11.7)	967 (12.9)	0.036
Chronic respiratory disease	132,901 (12.4)	1536 (20.5)	0.219
Ischemic heart disease	139,890 (13.1)	1323 (17.7)	0.127
Heart failure	50,586 (4.7)	492 (6.6)	0.080
Cerebrovascular disease	116,532 (10.9)	968 (12.9)	0.062
Chronic kidney disease	23,326 (2.2)	199 (2.7)	0.031
Cancer	41,531 (3.9)	307 (4.1)	0.011
Dementia	3900 (0.4)	37 (0.5)	0.020
Chronic liver disease	43,578 (4.1)	460 (6.1)	0.094
Tuberculosis	26,100 (2.4)	281 (3.8)	0.076
Prior influenza diagnosis	3788 (0.4)	51 (0.7)	0.046
Other non-influenza vaccination	9150 (0.9)	281 (3.8)	0.194
Pneumococcal vaccination	586 (0.1)	117 (1.6)	0.169
Sex: Female	469,422 (44.0)	3712 (49.6)	0.113
Sex: Male	447,734 (41.9)	3075 (41.1)	0.017
Sex: Unknown	150,705 (14.1)	698 (9.3)	0.149

**Note:** Continuous variables are presented as mean (SD); categorical variables are presented as *n* (%). The comparison was restricted to person-seasons classified as unvaccinated at the season-specific index date. “Subsequently vaccinated/censored” denotes person-seasons in which influenza vaccination occurred during follow-up and the primary analysis censored observation on the vaccination date. SMDs are presented as absolute values; values ≥0.10 indicate potentially meaningful baseline differences. SMD, standardized mean difference.

### Appendix A.6. Vaccination Timing Relative to the Season-Specific Index Date

To further characterize vaccination timing under the primary 14–365-day exposure definition, Table A5 summarizes the interval between influenza vaccination and the season-specific index date among vaccinated person-seasons, overall and by influenza season. Vaccination timing varied across study periods: more remote vaccination was particularly common in the two winter seasons, whereas summer-season vaccinations were concentrated mainly within 91–270 days before the index date. These descriptive data provide additional context for the progressively restricted 14–90-day, 14–180-day, and 14–270-day exposure-window sensitivity analyses reported in Table 7.

**Table A5:** Distribution of influenza vaccination timing before the season-specific index date among vaccinated person-seasons.

Season	Vaccinated Person-Seasons, *n*	14–90 Days, *n* (%)	91–180 Days, *n* (%)	181–270 Days, *n* (%)	271–365 Days, *n* (%)
2023 Summer	5123	927 (18.09)	1559 (30.43)	2517 (49.13)	120 (2.34)
2023 Winter	5943	1090 (18.34)	173 (2.91)	1260 (21.20)	3420 (57.55)
2024 Summer	6780	206 (3.04)	2801 (41.31)	3699 (54.56)	74 (1.09)
2024 Winter	7414	904 (12.19)	83 (1.12)	564 (7.61)	5863 (79.08)
2025 Summer	6280	225 (3.58)	2021 (32.18)	3943 (62.79)	91 (1.45)
Overall	31,540	3352 (10.63)	6637 (21.04)	11,983 (37.99)	9568 (30.34)

**Note:** Vaccination timing was calculated as the number of days between the recorded influenza vaccination date and the season-specific index date among person-seasons classified as vaccinated under the primary 14–365-day exposure definition. Percentages are calculated within each influenza season; the overall row is calculated across all 31,540 vaccinated person-seasons. The timing categories are mutually exclusive. Because lot-specific antigenic composition was not available for all vaccination records, these intervals describe vaccination recency and should not be interpreted as direct verification of season-specific vaccine formulation. Percentages may not sum to exactly 100% because of rounding.
