# Supplementary material for: Real-World Effectiveness of Influenza Vaccination Against Medically Attended Influenza and Severe Outcomes Among Older Adults in Yichang, China: A Multi-Season Population-Based Cohort Study
Source: Vaccines (Basel). 2026 Sep 28;14(10):858. doi: 10.3390/vaccines14100858 (PMC13709777; doi:10.3390/vaccines14100858)
Supplement: Supplementary file 1 [file vaccines-14-00858-s001.zip › Exploratory Product-Specific Estimates for Medically Attended Influenza.pdf]

Table S1. Exploratory Product-Specific Estimates for Medically Attended Influenza.

Comparison	Product category	Vaccinated person-seasons	Vaccinated events, n	HR (95% CI)	VE, % (95% CI)	p-value
Manufacturer	Manufacturer code 10	6,177	14	0.391 (0.222–0.689)	60.9 (31.1–77.8)	0.001
	Manufacturer code 12	3,306	6	0.308 (0.131–0.723)	69.2 (27.7–86.9)	0.007
	Manufacturer code 14	9,029	26	0.404 (0.265–0.616)	59.6 (38.4–73.5)	<0.001
	Manufacturer code 49	6,086	7	0.201 (0.088–0.462)	79.9 (53.8–91.2)	<0.001
	Manufacturer code 61	3,551	5	0.229 (0.089–0.592)	77.1 (40.8–91.1)	0.002
	Manufacturer code 79	1,861	5	0.472 (0.197–1.132)	52.8 (−13.2 – 80.3)	0.092
	Manufacturer code 78	1,280	2	0.284 (0.071–1.135)	71.6 (−13.5 – 92.9)	0.075
Valency	Quadrivalent	31,182	65	0.340 (0.261–0.443)	66.0 (55.7–73.9)	<0.001

Note: Product-specific analyses were exploratory and were conducted for medically attended influenza. Numeric manufacturer categories represent registry-specific manufacturer identifiers recorded in the source immunization data. Because no validated crosswalk between these internal codes and standardized manufacturer names was available, numeric codes were retained rather than retrospectively assigning potentially incorrect manufacturer identities; manufacturer names are shown only where they were explicitly recorded in the source data. Manufacturer- and valency-specific analyses were restricted to vaccinated person-seasons with available product information; therefore, product-specific counts do not necessarily sum to the total number of vaccinated person-seasons in the primary cohort. Some vaccination records lacked manufacturer and/or valency information. Estimates may be affected by differences in season, product availability, supply channel, recipient characteristics, and small numbers of vaccinated outcome events and should not be interpreted as head-to-head comparative effectiveness between manufacturers or vaccine products. No adjustment for multiple comparisons was applied; all product-specific estimates should therefore be interpreted as exploratory and hypothesis-generating. VE was calculated as $VE = (1 - HR) \times 100\%$. HR, hazard ratio; CI, confidence interval; VE, vaccine effectiveness.
